# ﻿A revision of a spring-active clade of *Prosoeca* Schiner, 1867 (Diptera, Nemestrinidae), keystone pollinators from the Greater Cape Floristic Region in South Africa, with descriptions of three new species

**DOI:** 10.3897/zookeys.1257.155954

**Published:** 2025-10-30

**Authors:** Genevieve L. Theron, Allan G. Ellis, John M. Midgley

**Affiliations:** 1 Department of Natural Sciences, KwaZulu-Natal Museum, Private Bag 9070, Pietermaritzburg, 3200, South Africa KwaZulu-Natal Museum Pietermaritzburg South Africa; 2 Current address: Biosystematics: Entomology, Plant Health and Protection, Agricultural Research Council, Private Bag X134, Queenswood 0121, South Africa Agricultural Research Council Queenswood South Africa; 3 Department of Botany and Zoology, Stellenbosch University, Private Bag X1, Matieland 7602, South Africa Stellenbosch University Matieland South Africa; 4 Department of Zoology and Entomology, Rhodes University, PO Box 94, Makhanda, 6140, South Africa Rhodes University Makhanda South Africa

**Keywords:** Afrotropics, long-tongued flies, Namaqualand, Nemestrinidae, new species, taxonomy

## Abstract

*Prosoeca* Schiner, 1867 is the most diverse genus of Nemestrinidae in Africa and is endemic to southern Africa. The 37 described species in this genus are all thought to be important pollinators for the plants that they visit. A recent phylogenetic study has shown that the diversity of this group is far larger than is formally recognised. Using of the phylogeny published by Theron et al. (2020), the monophyletic clade of six species from the Succulent Karoo that are on the wing during the spring flowering season are revised. The known species are redescribed and three new species are described: *Prosoeca ora***sp. nov.**, *P. aquilo***sp. nov.**, and *P. parva***sp. nov.** A lectotype is designated for *Prosoeca peringueyi* Lichtwardt, 1920. DNA barcodes have been generated for five species to aid molecular identification of species.

## ﻿Introduction

Nemestrinidae is a relatively small family of ~ 280 species in 17 extant genera, distributed across all continents except Antarctica (Bernardi 1973; Pujol-Luz and Lamas 2023). Two of the three subfamilies of Nemestrinidae occur in southern Africa, including 45 valid species in three genera within the Nemestrininae (Barraclough 2017; Barraclough et al. 2018; Theron et al. 2020; Barraclough and Colville 2024) and three species in three genera within Trichopsideinae (Barraclough 2006). These two subfamilies differ noticeably in the size of the proboscis, with the Nemestrininae usually having a well-developed proboscis (Bernardi 1973; Barraclough 2006), while it is vestigial or absent in the South African Trichopsideinae. South African species of Nemestrininae are thought to be nectar feeders and are often important pollinators (Manning and Goldblatt 1996, 1997, 2000; Potgieter and Edwards 2005; Barraclough 2006; Anderson and Johnson 2009; Newman et al. 2014), while Trichopsideinae species likely do not feed as adults (Bernardi 1973).

*Prosoeca* Schiner, 1867 (Diptera: Nemestrinidae: Nemestrininae) is the largest nemestrinid genus in Africa, with 37 valid species which are all thought to be endemic to southern Africa (Barraclough 2017; Barraclough et al. 2018; Theron et al. 2023). The vast majority of the accepted species are known from South Africa, where they have been recorded from seven of the nine biomes (Theron et al. 2023). A few species also occur in Namibia (e.g., *Prosoeca torquata* Theron, 2020 (Theron et al. 2020)) and Zimbabwe (*Prosoeca rhodesiensis* Bequaert, 1925 (Zamisa and Midgley 2022)). The taxonomic literature relating to *Prosoeca* is sporadic and disjointed, and a revision is needed to standardise descriptions (Barraclough 2017). The literature spans more than 200 years, since the description of *Prosoeca westermanii* (Wiedemann, 1821), originally as *Nemestrina* Latreille, 1809 (Wiedemann 1821). Schiner (1867) was the first to recognise the genus as distinct from *Nemestrina*. Important contributions include Loew (1858, 1860 - describing 4 species), Lichtwardt (1910, 1920 - 13 species), and Bezzi (1924 - 8 species). Numerous smaller contributions describing the remaining 12 species were published between 1828 and 2020. The type material is spread over at least 12 institutions in 11 countries, and in many cases, the type status of specimens is unclear. A morphological phylogeny of the genus has never been published and a molecular phylogeny showed that only 50% of the diversity has been described (Theron et al. 2023).

*Prosoeca* species are morphologically diverse, both in terms of size and colouration, often having elaborate patterning (Barraclough 2006). Notably, species exhibit remarkable differences in proboscis length (Manning and Goldblatt 2000; Theron 2021). *Prosoeca* species with long proboscides are often collected visiting long-tubed flowers in South Africa and many species are regarded as keystone pollinators (Manning and Goldblatt 1996, 2000; Potgieter and Edwards 2005; Anderson and Johnson 2009; Newman et al. 2014). In the Namaqualand region of the Succulent Karoo, a guild of 28 flower species were thought to rely exclusively on *Prosoeca peringueyi* for pollination (Manning and Goldblatt 1996). Recently, however, two new species of *Prosoeca* have been described that visit many of the same plant species in this region (Barraclough et al. 2018; Theron et al. 2020). The discovery of these additional species sheds new light on what were previously thought to be highly specialised plant-pollinator interactions and is facilitating a more complete understanding of the system as a whole (Pauw et al. 2020; Theron et al. 2020). Understanding pollination networks and pollinator redundancies within the system allows for better allocation of conservation resources. Despite the description of these species, further taxonomic work is needed. For example, Goldblatt and Manning (2007) showed that an undescribed *Prosoeca* species is the main pollinator of *Romulea syringodeoflora* de Vos, 1953, a narrow endemic in the Roggeveld region of the Succulent Karoo. Theron et al. (2023) highlighted the degree to which species diversity within *Prosoeca* has been underestimated, with at least 29 undescribed species identified.

The unclear status of type specimens, fragmented nature of the literature and low number of specimens of certain species has hindered the revision of *Prosoeca*. However, the recent production of a molecular phylogeny (Theron et al. 2023) allows smaller clades to be delimited and revised, advancing the taxonomy of the genus in tangible and pragmatic steps. Here we begin this process by revising the clade of spring-flying (early August to early November coinciding with the peak flowering) *Prosoeca* species from Namaqualand (clade D_4_ in Theron et al. 2023), providing modern, standardised descriptions for the known species, and describing three additional species.

## ﻿Materials and methods

### ﻿DNA extraction, amplification, and analysis

DNA barcodes were generated to aid in identification and to emphasise the validity of the new species described. Where possible, new barcodes were generated from fresh specimens of both sexes from different populations to supplement barcodes generated for previous studies (Table 1). *Moegistorhynchus longirostris* was included as an outgroup. Total DNA was extracted using the NucleoSpin Tissue Kit (Macherey-Nagel, Düren), according to the manufacturer’s instructions. The mitochondrial cytochrome c oxidase subunit I (COI) barcode region was amplified using the LCO1490 and HCO2198 (Folmer et al. 1994) primer pair. Each 25 μl reaction contained 1.5 mM MgCl_2_ in 1× PCR buffer (Invitrogen), 0.2 mM of each dNTP, 0.2 μM of each primer and 0.5 units of Taq polymerase (Invitrogen). The cycling protocol consisted of an initial denaturation step at 95 °C for 5 mins, followed by 35 cycles of denaturation at 95 °C for 45 s, annealing at 45 °C for 45 s, extension at 72 °C for 1.5 mins and a final extension of 5 mins at 72 °C. The PCR products were purified using the ExoSap protocol (Invitrogen), following the manufacturer’s instructions. PCR-products were sequenced bidirectionally using the ABI PRISM BigDye Terminator v. 3.1 Cycle Sequencing Kit and run on an ABI3130xl Genetic Analyzer.

**Table 1.** GenBank numbers of material used in the analysis, including source studies. Five of the six *Prosoeca* species treated are included and *M. longirostris* as an outgoup.

Species	Identifier code	GenBank number	Reference
*P. parva* sp. nov.	FG48	OP146648	Theron et al. 2023
*P. ora* sp. nov.	1410E07	PV704777	This study
	1410F01	PV704775	This study
	1410F02	PV704778	This study
*P. marinusi*	NV304	MT487555	Theron et al. 2020
	1410B02	PV704781	This study
	1410B03	PV704782	This study
	1410B04	PV704783	This study
*P. torquata*	V9	MT487549	Theron et al. 2020
	V4	MT487546	Theron et al. 2020
	NN1	MT487505	Theron et al. 2020
	NO3	MT487515	Theron et al. 2020
	V12	MT487552	Theron et al. 2020
	U1	MT487538	Theron et al. 2020
	KB16	PV704788	This study
	ST5	MT487536	Theron et al. 2020
	AH11	PV704786	This study
	3	PV704780	This study
	2	PV704779	This study
*P. peringueyi*	AP484	OP146623	Theron et al. 2023
	1410C03	PV704784	This study
	1410C01	PV704784	This study
	KB13	PV704787	This study
	VT13	MT487554	Theron et al. 2020
	NS10	PV704790	This study
	NGS-F06	PV704789	This study
	U5	MT487540	Theron et al. 2020
	NO4	MT487516	Theron et al. 2020
	NO6	MT487517	Theron et al. 2020
	U7	MT487542	Theron et al. 2020
	KB14	MT487504	Theron et al. 2020
*M. longirostris*	1410D08	PV704776	This study

The retrieved COI sequences were checked for inconsistencies and assembled using Geneious Prime v. 2025.0. Sequences were submitted to GenBank (Table 1). A consensus neighbour-joining tree, with 1000 bootstrap replicates, was constructed using the P-distance model in MEGA v. 11 (Tamura et al. 2021). Uncorrected p-distances were calculated with MEGA v. 11.

### ﻿Morphology

This revision is based on all available *Prosoeca* material (including types) from the Greater Cape Floristic Region, belonging to the clade D_4_ from Theron et al. (2023) (see Suppl. material 1 for summarised phylogeny). Museum name abbreviations follow Evenhuis (2020). Specimens were examined using a Zeiss Stemi 2000-C microscope. Morphological measurements were taken using a pair of digital callipers. Proboscis length was measured from the junction of the proboscis and the face to the tip of the un-extended proboscis. Body length was measured from the frons to the end of the abdomen, excluding the genitalia. Intertegular width was measured between the two wing bases. Wing length was measured from the tuft of hair at the wing base to the apex while the broadest part of the wing was measured by sliding a ruler across a photo of the wing in which *Sc* is approximately straight. The terminology for the description of morphology follows Cumming and Wood (2017) except we use the term pile instead of setulae as in Barraclough and Colville (2024). We note that the reference to facial area refers to both the face and the clypeus as they are difficult to differentiate, with the face being greatly reduced in Nemestrininae (Teskey 1981). Male terminalia dissections were placed in KOH and heated to accelerate maceration. Genitalia were rinsed in acetic acid, followed by water to stop maceration. Genitalia are stored in microvials filled with glycerol and associated with the relevant specimens in the collections. Images were compiled from stacked images taken with a Canon EOS 850D camera (Canon, Tokyo, Japan) using a modified set-up from Brecko et al. (2014). Images were stacked using Helicon Focus v. 8. Specimens from the following seven collections were examined for this revision:

**AMGS**Albany Museum, Makhanda, South Africa

**CDFA**California Department of Food and Agriculture, Sacramento, United States

**NHMUK**The Natural History Museum, London, United Kingdom

**NMSA**KwaZulu-Natal Museum, Pietermaritzburg, South Africa

**RMCA**Royal Museum for Central Africa, Tervuren, Belgium

**SAMC**Iziko South African Museum, Cape Town, South Africa

**SANC**South African National Collection of Insects, Pretoria, South Africa

**NMNW**National Museum of Namibia, Windhoek, Namibia

## ﻿Results

### ﻿DNA analysis

**Figure 1.**
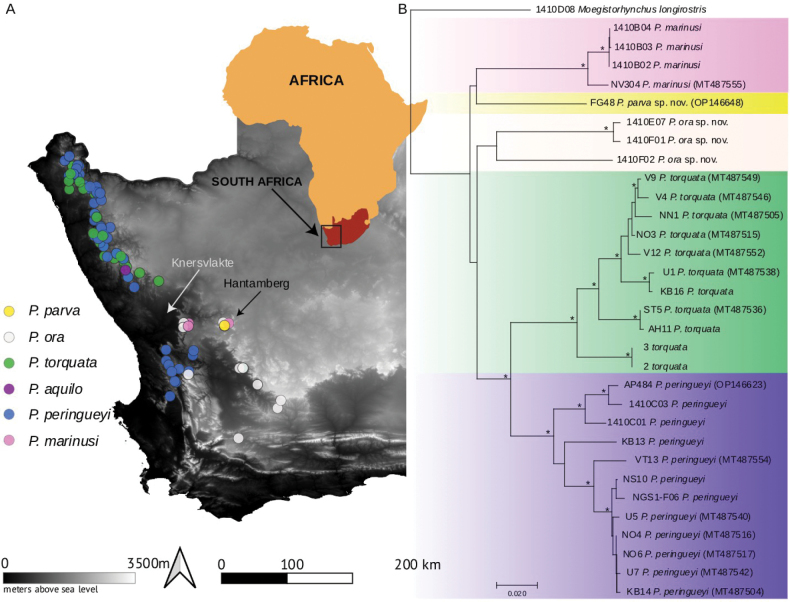
A. Map of species records from the Western and Northern Cape provinces of South Africa; B. Neighbour-joining tree of COI sequences for 5/6 species in the clade D_4_. Stars indicate >70% bootstrap support values for the node. GenBank numbers are in brackets next to the species name.

Thirty-one barcodes from specimens across the range (Fig. 1A) were used to form clusters representing five of the six species in this clade (Fig. 1B). The mean distance between species in this clade was 13.5% (range: 11.6%–15.2% between species) while the mean distance within species was 4.17% (range: 1.03%–8.05% between individuals) (Table 2). GenBank numbers for barcoded individuals are in Table 1. Many of the species in this clade show a degree of genetic structuring between populations but p-distances of these populations are all below the 11% threshold generally seen for different species in this clade.

**Table 2.** Mean p-distance within and between species barcodes. Grey highlighted numbers indicate mean within species p-distances. Unhighlighted numbers indicate between species mean p-distances.

	*P. ora* sp. nov.	*M. longirostris*	*P. torquata*	*P. marinusi*	*P. peringueyi*	*P. parva* sp. nov.
*P. ora* sp. nov	0.0805					
*M. longirostris*	0.164	n/c				
*P. torquata*	0.146	0.171	0.0381			
*P. marinusi*	0.141	0.156	0.152	0.0103		
*P. peringueyi*	0.139	0.161	0.119	0.142	0.0378	
*P. parva* sp. nov.	0.120	0.143	0.147	0.116	0.131	n/c

#### ﻿Clade D_4_ diagnosis (Figs 1–8)

This clade can be distinguished from all other clades of *Prosoeca* by the combination of upturned longitudinal veins *R_2+3_*, *R_4_*, *R_5_*, *M_1_*, and *M_2_* (Fig. 5) (relatively straight in clades D_1_, D_2_, and D_3_), a pale grey pruinescent border on the scutum and scutellum, and a dark black posterior margin on the scutellum (Fig. 3) (border absent or incomplete in other clades). All species in this clade can be found on the wing in the winter-rainfall regions of the Northern Cape and Western Cape of South Africa during the spring (Fig. 1).

#### ﻿Clade D_4_ notes

Body small- to large-sized (length 8–22 mm), abdomen with dark medial vitta, except in *P. parva* sp. nov. (Fig. 3), wing with isolated (may be joined to anterior infuscation, especially in *P. peringueyi*) infuscation on fork of *R_4_* and *R_5_* (indistinct in *P. parva* sp. nov.) (Fig. 4). The Greater Cape Floristic Region where these species were found forms a complex matrix of Succulent Karoo and Fynbos biomes, and most species in this clade are likely to be found in both biomes.

In *Prosoeca*, the pruinescence is largely responsible for the colour patterning, thus specimens that have been exposed to moisture or excessive ethyl acetate may easily lose the patterning, making identification difficult and in extreme cases impossible.

### ﻿Key to species of clade D_4_

**Table:** 

1	Proboscis much longer than body length (Fig. 4A, B), distinct vittae and/or patterning present on the thorax (Fig. 3A–C)	**2**
–	Proboscis shorter or of similar length to the body (Fig. 4E, F), vittae and patterning absent/indistinct apart from grey border on the lateral margins of the thorax (Fig. 3D, F)	**4**
2	Wings with anterior infuscation never strikingly distinct from the paler posterior margin (Fig. 5B), abdominal tergites with medial vitta often present (may not be visible in some individuals) without sub-lateral markings, (Fig. 3B)	***P. marinusi* Barraclough, 2018**
–	Wings with dark brown anterior infuscation strikingly distinct from posterior hyaline area (Fig. 5A, C), abdominal tergites with medial vitta and irregular sublateral patterning (Fig. 3A, C)	**3**
3	Proboscis 14–24 mm, with a white band of pile anterior on the thorax, sublateral vittae present on the thorax but indistinct (Fig. 3A), cross-vein joining *R_4_* and *R_2+3_* never present (Fig. 5A)	***P. torquata* Theron, 2020**
–	Proboscis 23–35 mm, without dense white band of pile anterior on the thorax, distinct dark black sublateral vittae on the thorax (Fig. 3C), cross-vein joining *R_4_* and *R_2+3_* present in northern populations where they are sympatric with *P. torquata* but cross-vein absent in populations south of the Knersvlakte (Fig. 5C)	***P. peringueyi* Lichtwardt, 1920**
4	Wings with slight but distinct anterior curved flexure in males, dark brown anterior infuscation distinct from the hyaline area (female unknown) (Fig. 5D), facial area and frons with sparse white pile (Fig. 6D)	***P. aquilo* sp. nov.**
–	Wing margin close to straight without distinct anterior curved flexure in males, pale anterior infuscation somewhat distinct from the hyaline area (Fig. 5E, F), facial area and frons with dense white pile (Fig. 6E, F)	**5**
5	Abdomen with dull grey pruinescent patterns (Fig. 3F), wings almost entirely hyaline (Fig. 5F), all femora noticeably darker than tibiae and tarsi, hind femora dark black with long, sparse, black pile (Fig. 4F), species of small size: intertegular width 3–4 mm, proboscis 4–6 mm	***P. parva* sp. nov.**
–	Abdomen with regular pale grey, black and brown pruinescence patterning (Fig. 3E, wings with pale infuscation on anterior ¼–½ (Fig. 5E), ventral side of fore femur similar in colour to tibia and tarsi, hind femur dark red-brown with long, sparse, pale pile proximally and short, dense golden pile ventrally (Fig. 4E), species of variable size: intertegular width 4–8 mm, proboscis 6–11 mm	***P. ora* sp. nov.**

### ﻿Taxonomy

#### ﻿Genus Prosoeca

Taxon classificationAnimaliaDipteraNemestrinidae

Schiner, 1867

80C7E568-43AD-5DB0-8A43-42A3864073FD

Prosoeca Schiner, 1867; Bezzi 1924; Bernardi 1973; Bowden 1980; Barraclough 2006.

##### Type species.

*Nemestrina westermanni* (Weidemann, 1821), by original designation.

#### ﻿ Prosoeca torquata

Taxon classificationAnimaliaDipteraNemestrinidae

Theron, 2020

76DBCF73-2749-50AE-A12E-CD89888A8DAF

Figs 1, 3A, 4A, 5A, 6A, 7A, B

Prosoeca torquata Theron, 2020: 29.

##### Type material examined.

***Holotype***: South Africa • ♂ Northern Cape, Steinkopf, Kosiesberg; −29.12631, 17.55686; 3 Aug 2019; F. Grenier leg.; NMSA-DIP 166602; NMSA. ***Paratypes***: South Africa • 1 ♀ N. Cape, Steinkopf, Kosiesberg; -29.12631, 17.55686; 3 Aug. 2019; T. van der Niet leg.; NMSA-DIP 166605; NMSA. • 1 ♂ 3 ♀♀ Cape, Steinkopf, Kosiesberg; -29.12631, 17.55686; 3 Aug. 2019; F. Grenier leg.; NMSA-DIP 166602–166606; NMSA. • 3 ♂♂ Northern Cape, Springbok, Naries; -29.69043, 17.66491; 4 Aug. 2019; F. Grenier leg.; NMSA-DIP 166607–166609; NMSA. • 1 ♀ 1 ♂ Steinkopf, Kosiesberg; -29.12631, 17.55686; 3 Aug. 2019; T. van der Niet leg.; RMCA ENT 13300, RMCA-ENT 13301; RMCA.

##### Other material examined.

South Africa • 1 ♂ Northern Cape: Kamiesberg: Leliefontein; 19 Oct. 2008; 34 visiting *Satyrium erectum*; T. van der Niet leg.; NMSA-DIP 219367; NMSA. • 1 ♀ Northern Cape: Nama Khoi: Naries; 29.701°S, 17.665°E; Spring 2009; T. van der Niet leg.; *Satyrium erectum*; NMSA-DIP 219347; NMSA. • 1 ♂ Northern Cape: Steinkopf: Kosiesberg; 26 Aug 2016; Bruce Anderson leg; NMSA-DIP 221846; NMSA • 2 ♂♂ 1 ♀ Northern Cape: Namaqualand: Kamiesberg: 2 km S of Leliefontein; 17 Sep. 1992; F.W. and S.K. Gess leg.; On pink flowers of Pelargonium cf. incrassatum; AMGS-ENT 101691–101693 AMGS. • 2 ♂♂ 2 ♀♀ Northern Cape: Namaqualand: Kamiesberg: 2 km S of Leliefontein; 12 Sep. 1992; F.W. and S.K. Gess leg.; Flowers: *Hesperantha paucifolia* (Iridaceae); AMGS-ENT 101694–101697; AMGS. • 1 ♂ Northern Cape: Namaqualand: Kamiesberg: 2 km S of Leliefontein; 13 Sep. 1992; F.W. and S.K. Gess leg.; On deep pink flowers of *Pelargonium* sp. (Geraniaceae); AMGS-ENT 101698; AMGS. • 1 ♂ Northern Cape: Namaqualand: Kamiesberg: 2 km S of Leliefontein; 17 Sep. 1992; F.W. and S.K. Gess leg.; AMGS-ENT 101700; AMGS. • 1 ♂ Northern Cape: Namaqualand: Kamiesberg: 2 km S of Leliefontein; 12 Sep. 1992; F.W. and S.K. Gess leg.; AMGS-ENT 101699; AMGS. • 1 ♂ 1 ♀ Namaqualand: Bowesdorp; SA Museum staff leg.; Sep. 1941; SAM-DIP A009011; SAMC. NAMIBIA • 1 ♀ Namuskluft 88: !NamiNus constituency (on label as Luderitz); SE 2710 Dd; 20–22 Sep. 1973; H14602; NMNW.

##### Diagnosis

(adapted from Theron et al. 2020). Medium- to large-sized (length 12–21 mm), dark body, with intricate patterns on the abdomen, dark brown legs, proboscis length 1.10 ± 0.02× the length of the body (range of un-extended proboscis length 14–24 mm), and patterned smoky brown infuscation on the anterior 1/2 of the wing. *Prosoeca torquata*, *P. marinusi* Barraclough, 2018 and *P. peringueyi* Lichtwardt, 1920 can be distinguished from all other species in the clade by their proboscis which is longer than the length of their bodies, in contrast to *P. ora* sp. nov., *P. aquilo* sp. nov. and *P. parva* sp. nov. that have a proboscis shorter than the length of their body. *Prosoeca torquata* and *P. peringueyi* both have a distinct dark smoky brown wing anterior, abruptly becoming hyaline on the posterior section (Fig. 5A, C). *Prosoeca torquata* differs most noticeably from *P. peringueyi* and *P. marinusi*, by the presence of a white band of pile on the anterior of the thorax and white pile on the face (Figs 3A, 6A), which is largely lacking in the latter two species. Additionally, *P. torquata* has a darker thorax than *P. peringueyi*. *Prosoeca torquata* generally has a proboscis that is only slightly longer than the body, in comparison to *P. marinusi* and *P. peringueyi*, which both have a proboscis substantially longer than the body. *Prosoeca torquata* can be found north of the Knersvlakte to the southern part of Namibia.

##### Redescription.

**Male.** Body length: mean 15.7 mm; range 12–21 mm (*n* = 42). Intertegular width: mean 6.1 mm; range 4–8 mm (*n* = 12). Proboscis length: mean 17.8 mm; range 14–24 mm (*n* = 33). Wing length: mean 16.5 mm; range 12–19 mm (*n* = 14).

***Head***. (Figs 4A, 6A) Ground colour generally red-brown to dark brown or black. Ocellar tubercle somewhat bulbous and developed, just evident above upper eye margin in profile, with dense silvery pruinescence and long black pile; width between eyes at the anterior ocellus 2.2–2.5× the length of the anterior ocellus; anterior ocellus separated from posterior ocelli by shallow transverse groove. Frons trapezoid; width anterior to ocellar tubercle 0.6× the width above antennal insertions; slightly to moderately swollen between antennal insertions and anterior ocellus; swelling recedes strongly towards eye margin; pruinescence relatively dense, silver to brown; pile generally absent. Antenna with scape 1.2–1.5× length of pedicel; first flagellomere longer than the length of scape + pedicel; style subequal to or longer than scape + pedicel + flagellomere 1; ground colour transitioning to a darker hue, on the proximal side of the first flagellomere, style darker than remainder of antenna; scape, pedicel and flagellomere 1 with irregular silver to brown pruinescence; pedicel with mostly elongate black pile, flagellomere 1 with short black pile basally on the dorsal side. Facial area bulbous in profile, with horizontal groove present; sublaterally irregular yellow-brown; with silver to brown pruinescence, but pruinescence largely absent from medial section of face; pile mostly white, elongate, dense, more dense than on frons. Gena with pile a mixture of black and white to pale yellow, elongate, and dense, forming the beard. Proboscis 0.9–1.3× the length of the body, black with dorsal part of basal 1/3–1/2 brown. Palpus with first segment significantly longer and much wider than the second segment; colour generally dark brown to black; pile longer on the first segment.

***Thorax*** (Figs 3A, 4A). Scutum dark grey to black; pruinescence mostly brown, with pale grey to silvery pruinescence along the lateral sides of the scutum, joining on scutellum; median and paired sublateral vittae evident but never striking, or indistinct; pile mostly black, sparse, shorter than the pile posterior on the scutellum; pile on postpronotal lobe and anterodorsal region of scutum white, dense forming broad collar; postalar callus with black pile dorsally, ventral side with a tuft of white pile. Scutellum with distinct darker central dot or diamond; anterior margin covered by silver to brown pruinescence; posterior margin with a dark, black border; pile on disc of scutellum black, relatively long, sparse compared to scutum; pile along posterior margin elongate, a mixture of black and pale, white or yellow, dense compared to disc of scutellum; with some pale yellow to golden pile laterally. Pleuron mostly blackish; with silver pruinescence, sparser than on scutum; pile generally a mixture of black and white to yellow, relatively long, of intermediate density; most dense and elongate in two tufts, ventral and anterior to the base of the wing and between postalar callus and posterior spiracle; tuft of pile anterior to wing base directed posteriorly, mostly white to golden with some black pile; tuft of pile on katatergite directed posteriorly, black and golden; katepimeron with pile absent, or sparse elongate white pile. ***Legs***. Coxae yellow brown to dark brown; with pile mostly off-white to golden, elongate, dense. Trochanters mostly blackish, with some yellow-brown colouring; pile short, very sparse. Femora yellow-brown, with dark marking on dorsal side of the distal end present; pile mostly black, mostly short, dense, but with elongate pile dorsally on proximal 1/3–1/2; ventral pile typically longer, sparse; hind femur with short pile more evenly distributed than on fore and mid femora. Tibiae yellow-brown to dark brown; with dense, short, pale pile and sparse, elongate, darker pile (sometimes only short pile present), most dense on hind tibia. Tarsi red-brown to dark brown, hind tarsi tend to be darker. ***Wings*** (Fig. 5A). Shape relatively slender; broadest just basal to termination of *CuP* on posterior margin; alula broad; costal margin close to straight, without distinct anteriorly curved flexure; *Sc* termination on *C* aligned with termination of *M_4_* on posterior margin of wing; *R_1_* terminated closer to *R_2+3_* than to *Sc*; termination of *Sc* and *R_1_* well separated; short appendix just beyond fork on *R_4+5_* sometimes present; cross vein between *M_1_* and *M_2_* absent; cross vein between *R_4_* and *R_2+3_* absent; *R_1_* relatively straight; *R_4_* and *R_5_* deeply bowing upward; *M_1_* and *M_2_* gently bowing upward; cell *cua* open at margin; *CuA* and *CuP* well separated. Dark marking on *R_1_* positioned just basal to humeral cross vein; membrane with smoky brown infuscation; appearing darker on anterior 1/2–1/3 of wing; posterior region of wing mostly hyaline; isolated darker patches distinct in hyaline region; the distinction between brown infuscation and hyaline membrane sharply delineated. Tuft of pile on base of wing white. Haltere with pale brown to yellow stalk; bulb dark brown.

***Abdomen*** (Fig. 3A). Colour of abdomen generally red-brown; T_2_ with posterior margin stout and relatively broad; abdomen tapering abruptly after T_3_. Tergites with brown or silvery pruinescence; membrane between T_1_ and T_2_ with silvery to brown pruinescence; medial brown pruinescent vitta distinct, extending from the posterior margin of T_1_ to terminalia, usually not covering the full length of each tergite; paired sublateral markings of indistinct shape, on T_2_–T_4_. Pile on tergites mostly black, both long and short, of intermediate density; along anterior margins of T_2_ white to pale yellow, elongate, sparse; posterolateral pile on T_2_–T_4_ black, elongate, dense; T_5_ with pile along lateral margins more evenly distributed than that of T_2_–T_4_. Sternites typically paler than tergites; pale reddish brown; pruinescence silvery, relatively sparse. Sternites with pile a mixture of black and white, mostly short, sparse; pile on S_1_–S_3_ intermixed, long, black or white; pile on membrane adjacent to lateral margins of S_2_–S_4_ typically with profuse, decumbent, elongate, white pile.

***Genitalia*** (Fig. 7A, B). Hypandrium triangular in shape; relatively narrow, tapering gradually towards the apex; laterally straight sided; 1.8× longer than basal width; with apical 1/5 projecting past the top of the gonocoxites. Hypandrium vestiture short, sparse, on apical 1/3. Gonocoxite apical 1/2 parallel sided; gonocoxites widest in basal 1/2, narrowing apically; rounded apically. Gonocoxite vestiture on the lateral 1/2 of apical 1/2 long, laterally projecting. Gonostylus narrowed medially; with globular apical section. Phallus near parallel sided; narrowing apically.

**Female.** Same as male, except for genitalia dimorphism and the following characters: ***Head***. Ocellar tubercle width between eyes at the anterior ocellus 3–3.5× the length of the anterior ocellus. Frons width anterior to ocellar tubercle 0.8× the width above antennal insertions.

##### Geographical distribution.

*Prosoeca torquata* occurs between Namuskluft in Namibia (single NMNW specimen) in the north and the Knersvlakte region in the Northern Cape Province at Uilklip in the south and is generally abundant when host plants are flowering (Fig. 1).

##### Biology.

The *P. peringueyi* complex, including *P. torquata*, is known to visit many plant species (Manning and Goldblatt 1996, 2000; Pauw et al. 2020) in both Fynbos and Succulent Karoo biomes. It is, however, not precisely known whether *P. peringueyi* and *P. torquata* are pollinators of the same suite of plants, or if they partition floral resources. *Prosoeca torquata* is known with some certainty, from specimen labels, to visit *Lapeirousia silenoides*, *Pelargonium echinatum*, *Pelargonium crithmifolium*, *Satyrium erectum*, *Pelargonium incrassatum*, and *Hesperantha pauciflora*. Published data that do not refer to vouchered specimens are of unclear value, as they may refer to *P. torquata* or *P. peringueyi*.

#### ﻿ Prosoeca marinusi

Taxon classificationAnimaliaDipteraNemestrinidae

Barraclough, 2018

10246222-AE72-5551-832D-9E14D669CBB9

**Figure 2.**
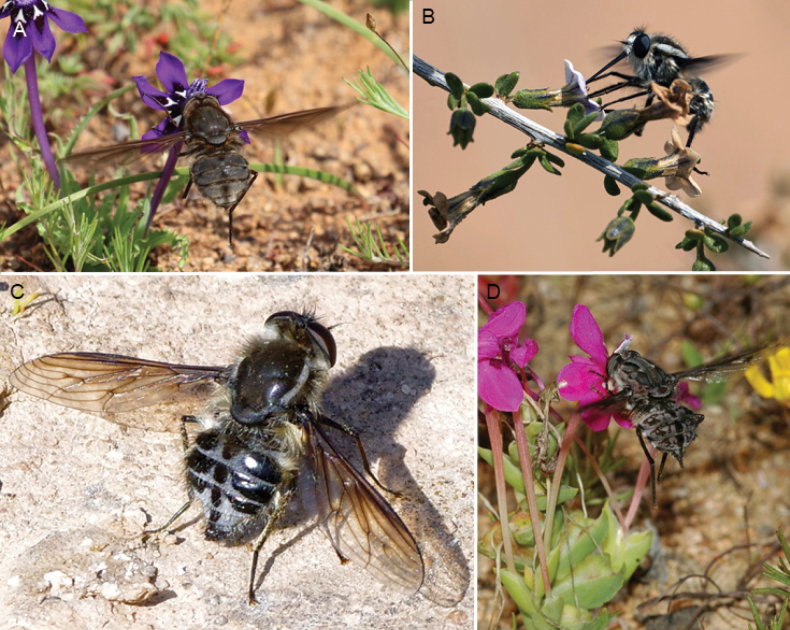
Photographs of *in situ* adults. A. *Prosoeca marinusi* visiting *Lapeirousia oreogena*; B. *Prosoeca ora* sp. nov. visiting *Lycium* sp.; C. *Prosoeca ora* sp. nov. resting; D. *Prosoeca peringueyi* visiting *Lapeirousia silenoides*.

**Figure 3.**
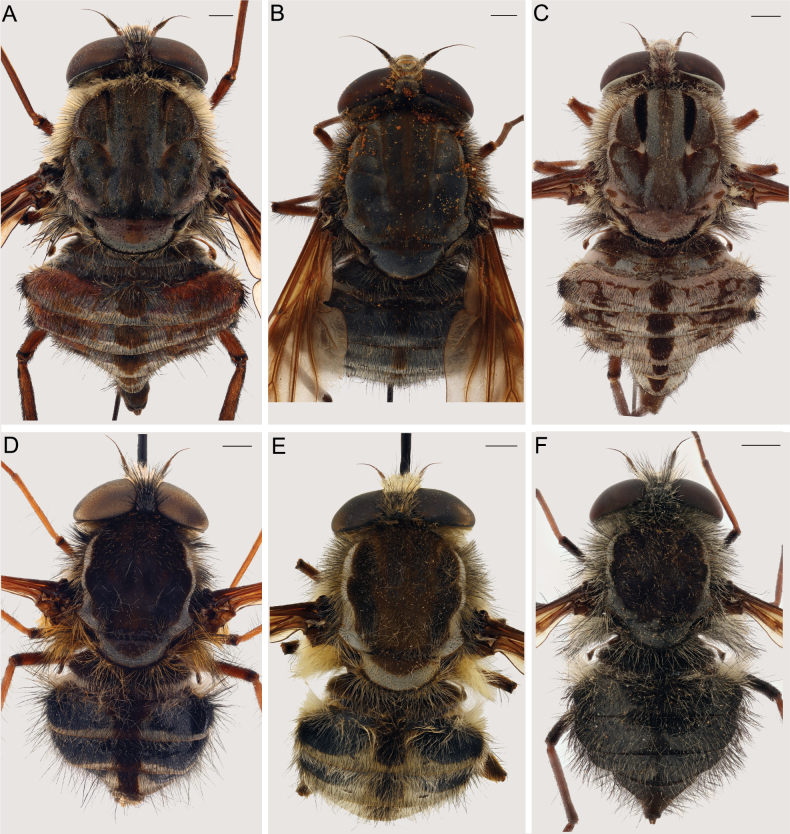
Dorsal view of A. *Prosoeca torquata*NMSA-DIP 166609; B. *P. marinusi*NMSA-DIP 205673; C. *P. peringueyi*NMSA-DIP 76251; D. *P. aquilo* sp. nov. RMCA-ENT 000056700; E. *P. ora* sp. nov. NMSA-DIP 219345; F. *P. parva* sp. nov. NMSA-DIP 76671. Scale bars: 1 mm.

**Figure 4.**
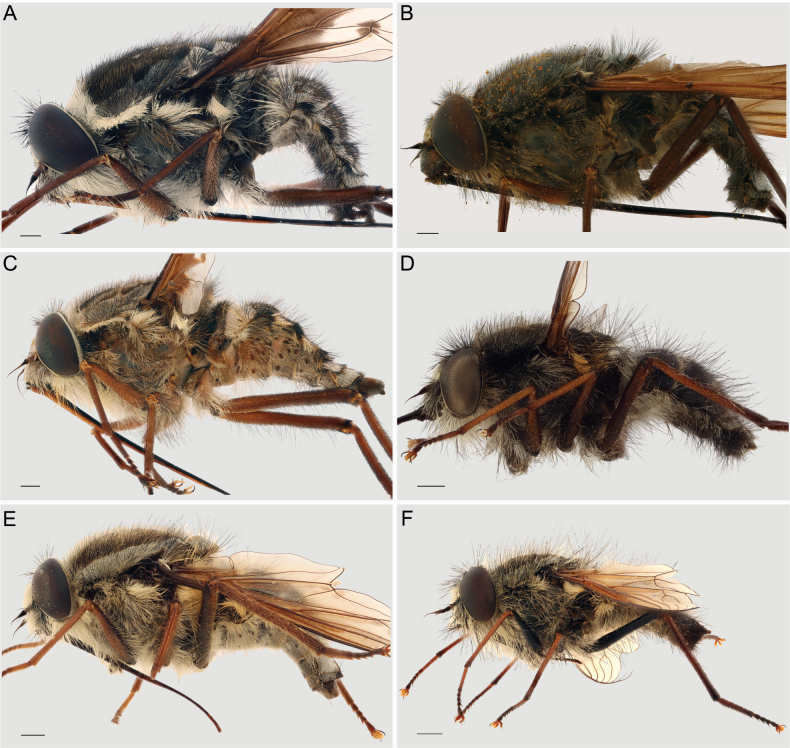
Lateral view of A. *Prosoeca torquata*NMSA-DIP 166609; B. *P. marinusi*NMSA-DIP 205673; C. *P. peringueyi*NMSA-DIP 76251; D. *P. aquilo* sp. nov. RMCA-ENT 000056700; E. *P. ora* sp. nov. NMSA-DIP 219345; F. *P. parva* sp. nov. NMSA-DIP 76671. Scale bars: 1 mm.

**Figure 5.**
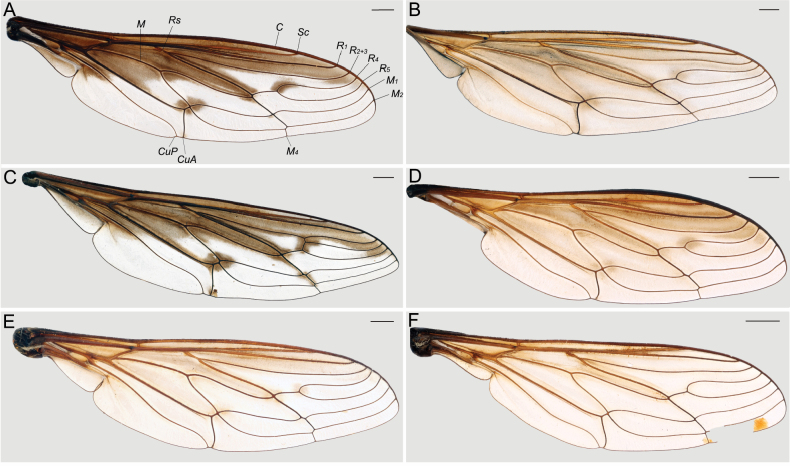
Wings A. ♂ *Prosoeca torquata*NMSA-DIP 221846; B. ♂ *P. marinusi*NMSA-DIP 52484; C. ♀ *P. peringueyi*NMSA-DIP 219355; D. ♂ *P. aquilo* sp. nov. NMSA-DIP 79006; E. ♀ *P. ora* sp. nov. NMSA-DIP 79018; F. ♂ *P. parva* sp. nov. NMSA-DIP 76671. Abbreviations: *C*, costal vein; *CuA*, anterior branch of cubital vein; *CuP*, posterior branch of cubital vein; *M*, medial vein; *M_1_*, first branch of media; *M_2_*, second branch of media; *M_4_*, fourth branch of media; *R_1_*, anterior branch of radius; *R_2+3_*, second branch of radius; *R_4_*, upper branch of third branch of radius; *R_5_*, lower branch of third branch of radius; *Rs*, radial sector; *Sc*, subcostal vein. Scale bars: 1 mm.

**Figure 6.**
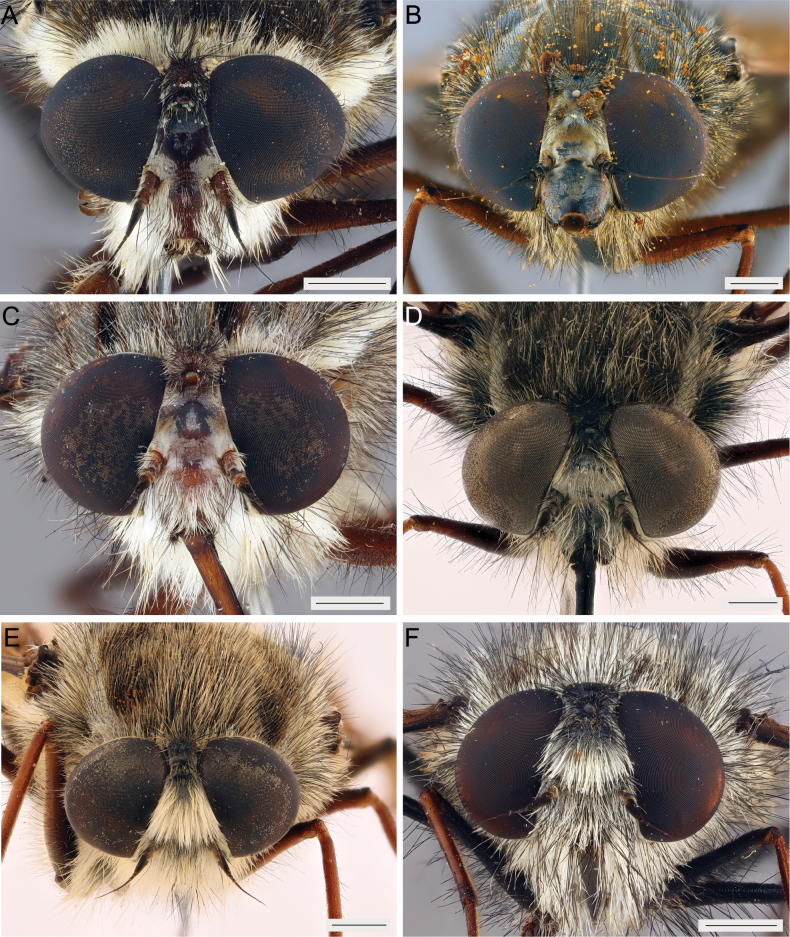
Frontal views A. *Prosoeca torquata*NMSA-DIP 221767; B. *P. marinusi*NMSA-DIP 205673; C. *P. peringueyi*NMSA-DIP 52845; D. *P. aquilo* sp. nov. NMSA-DIP 79002; E. *P. ora* sp. nov. NMSA-DIP 54396; F. *P. parva* sp. nov. NMSA-DIP 76671. Scale bars: 1 mm.

**Figure 7.**
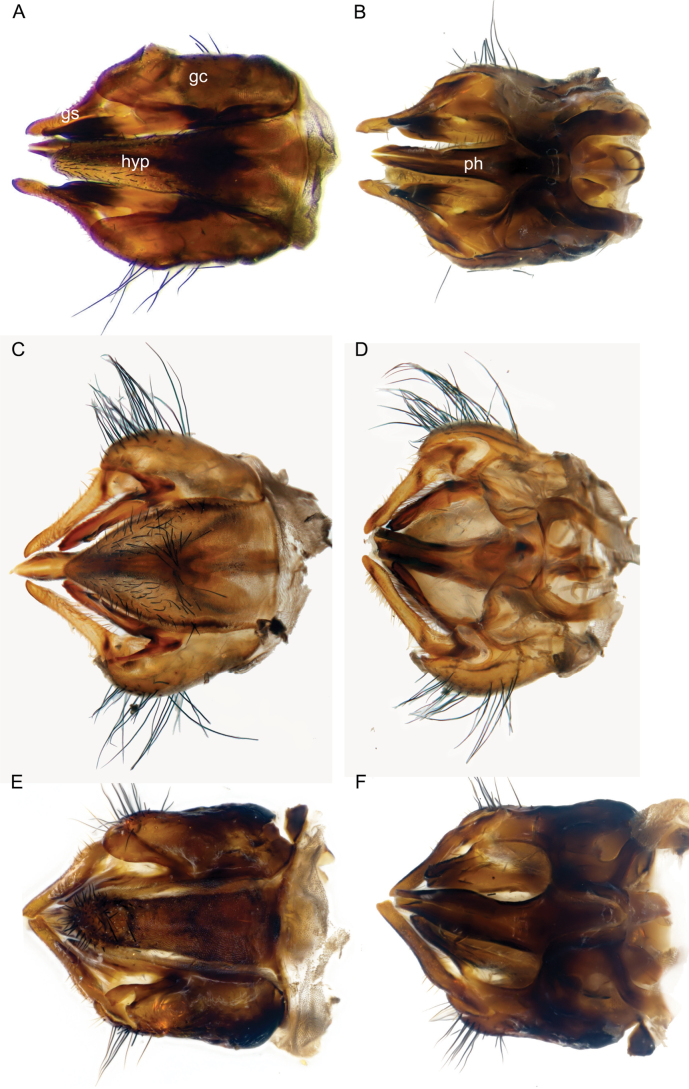
Genitalia A, B. *Prosoeca torquata*; C, D. *P. marinusi*; E, F. *P. peringueyi*. A, C, E. dorsal view; B, D, F. ventral view. Abbreviations: gc, gonocoxite; gs, gonostylus; hyp, hypandrium; ph, phallus.

Figs 1, 2A, 3B, 4B, 5B, 6B, 7C, D

Prosoeca marinusi Barraclough, 2018: 412.

##### Type material examined.

***Holotype***: South Africa: • 1 ♂ Northern Cape: Nieuwoudtville Reserve; 3119 AC; 5 Aug. 1988; K. Steiner leg.; 2139; Host Plants, *Lapeirousia oreogena*; Time: 1030–1200; SAM-DIP A012376; SAMC. ***Paratypes***: South Africa: • 3 ♂♂ 1 ♀ Northern Cape: Nieuwoudtville Reserve; 3119 AC; 5 Aug. 1988; K. Steiner leg.; Host plant *Lapeirousia oreogena*; Time 1030–1200; 2146, 2147, 2141, 2144; SAM-DIP A012376; SAMC. • 1 ♂ Northern Cape: Glen Lyon; 3119 NC; 26 Aug. 1990; K. Steiner leg.; 2664; *Nemesia cheiranthus*; SAM-DIP A012379; SAMC. • 1 ♀ Northern Cape: Nieuwoudtville Flower Res.; 3119 AC; 20 Aug. 1986; K. Steiner; 1366; *Lapeirousia oreogena* 1602; SAM-DIP A012378; SAMC. • 2 ♀♀ Northern Cape, Nieuwoudtville Flower Res.; 3119 AC; 19 Aug. 1986; K. Steiner leg.; 1364 *Lapeirousia oreogena* 1408, 1365 *Lapeirousia oreogena* 1430; SAM-DIP A012377; SAMC. • 3 ♂♂ 1 ♀ Northern Cape: Nieuwoudtville Reserve, 3119 AC; 5 Aug. 1988; K. Steiner leg.; 2140, 2145, 2142, 2138; SAM-DIP A01S2376; SAMC. • 3 ♂♂ Northern Cape: Nieuwoudtville: Farm Melkbosfontein; 31°21.12'S, 19°10.22'E; elev. 756 m; *Lapeirousia oreogena*; Melin A, Colville JF, Krenn H & Karolyl F leg.; 23–25 Aug. 2014; 2143, 2136, 2137; SAM-DIP A012376; SAMC. • 1 ♂ Northern Cape: Nieuwoudtville Flower Reserve; 2 Aug. 1984; K. Steiner leg.; SAM-DIP A012384; SAMC.

##### Other material examined.

South Africa: • 1 ♂ 2 ♀♀ Northern Cape Province: Nieuwoudtville: Farm Melkbosfontein; 31°21.12'S, 19°10.22'E; elev. 756 m; *Lapeirousia oreogena*; Melin A, Colville JF, Krenn H & Karolyl F leg.; 23–25 Aug. 2014; SAM-DIP A015475, SAM-DIP A015476, SAM-DIP A015488; SAMC. • 1 ♀ Northern Cape: Nieuwoudtville: Hantamsberg Nat Botanical Garden; 735 m Spider Trail area; 31°23.51'S, 19°08.24'E; J&A Londt leg.; 11 Sep. 2012; NMSA-DIP 205675; NMSA. • 1 ♂ Northern Cape: Nieuwoudtville: Hantamsberg Nat Botanical Garden; Rocky ridge; 31°23.51'S, 19°08.24'E; J&A Londt leg.; 11 Sep 2012; NMSA-DIP 205673; NMSA. • 1 ♂ 1 ♀ Northern Cape: Nieuwoudtville: Hantamsberg Botanical Garden; 31.39815°S, 19.14107°E; Steven Johnson leg.; 25–28 Aug 2019; NMSA-DIP 219352, NMSA-DIP 219353; NMSA. • 2 ♂♂ Northern Cape: Calvinia: Hantamsberg; 3 Sep. 1994; 31; NMSA-DIP 079030, NMSA-DIP 52483 [handwriting of Dr. J. Manning]; NMSA. • 1 ♂ Northern Cape: Calvinia: Hantamsberg; 3 Nov. 1994; J. Manning leg.; NMSA-DIP 52484; NMSA. • 1 ♀ Northern Cape: 5 km N Nieuwoudtville; 3119AC; 5 Sep.1981; J. Londt, L. Schoeman and B. Stuckenberg leg.; W. Mountain Karoo; NMSA-DIP 42755; NMSA. • 1 ♂ Northern Cape: Nieuwoudtville; 23 Aug. 1993; on *Lapeirousia oreogena*; 2; NMSA-DIP 51662; NMSA. • 1 ♂ Northern Cape, Nieuwoudtville; 23 Aug. 1993; on *Lapeirousia oreogena*, 3, NMSA-DIP 76674; NMSA. • 1 ♀ Northern Cape: Nieuwoudtville; 32; 4 Sep. 1994; on *Lapeirousia oreogena*; NMSA-DIP 52474 [handwriting of Dr. J. Manning]; NMSA. • 1 ♀ Northern Cape: Nieuwoudtville Dist.: Oorlogskloof; Sep. 1992; J. Manning leg.; on *Lapeirousia jacquinii*; NMSA-DIP 54391; NMSA. • 1 ♀ Northern Cape: 13.5 km N Nieuwoudtville; 31.16°S, 19.08°E; 18 Sep. 2007; C. Eardley leg.; SANC.

##### Diagnosis.

Large-sized (length 14–22 mm), grey body with paler medial and paired sub-lateral vittae on the thorax and darker medial vitta present on the abdomen, scutellum with black posterior border, legs dark brown, proboscis length 2.19 ± 0.05× the length of the body (range of un-extended proboscis length 31– 46 mm), wings with smoky brown infuscation on the anterior 2/3 with no hyaline section of the wing. *Prosoeca marinusi*, *P. torquata*, and *P. peringueyi* can be distinguished from all other species in the clade by their proboscis which is longer than the length of their bodies, in contrast to *P. ora* sp. nov., *P. aquilo* sp. nov., and *P. parva* sp. nov. that have a proboscis shorter than the length of their body. *Prosoeca marinusi* has a distinctly darker posterior section of the wing that is never hyaline, compared to all other species in the clade (Fig. 5B). *Prosoeca marinusi* differs from *P. peringueyi* and *P. torquata*, the other long-proboscid species in the clade, by having only a medial vitta on the abdomen (Fig. 3B), with the sublateral patterning present in *P. peringueyi* (Fig. 3C) and absent in *P. torquata* (Fig. 3A). The hypandrium of *P. marinusi* is substantially broader basally than that of *P. peringueyi* or *P. torquata*. *Prosoeca marinusi* is a narrow endemic occurring in the Nieuwoudtville and Calvinia areas.

Some individuals may appear very dark, particularly on the abdomen, while others have a more subtle grey colouration. This dark colouration can largely be attributed to an abundance of dark pile that is less profuse and mixed with pale pile in paler individuals.

##### Redescription.

**Male**. Body length: mean 18 mm; range 14–22 mm (*n* = 19). Intertegular width: mean 7.3 mm; range 6–9 mm (*n* = 19). Proboscis length: mean 39.2 mm; range 31–46 mm (*n* = 19). Wing length: mean 21.8 mm; range 19–24 mm (*n* = 17).

***Head***. (Figs 4B, 6B) Ground colour generally grey to black. Ocellar tubercle somewhat bulbous and developed, just evident above the upper eye margin in profile, with dense silvery pruinescence; width between eyes at the anterior ocellus 4.3–4.8× the length of the anterior ocellus; anterior ocellus separated from posterior ocelli by shallow transverse groove; pile generally long (shorter than that of *P. torquata* and *P. ora*), black. Frons trapezoid; width anterior to ocellar tubercle 0.8× the width above antennal insertions; slightly to moderately swollen between antennal insertions and anterior ocellus; swelling recedes strongly towards eye margin; pruinescence relatively dense, brownish; pile black and white, sparse along lateral margins towards antennal insertions. Antenna with scape 1.5–2× length of pedicel; first flagellomere subequal to the length of scape + pedicel; style longer than scape + pedicel + flagellomere 1; ground colour transitioning to a darker hue on the scape, style paler than remainder of antenna; scape, pedicel and flagellomere 1 with irregular silver to brown pruinescence; pedicel with both elongate and short, black and white pile, flagellomere 1 with short black pile basally on the dorsal side. Facial area bulbous in profile, with horizontal groove present; sublaterally irregular yellow-brown; with sparse silver to brown pruinescence, evenly distributed across face; pile brown to black, elongate, sparse, less dense than on frons. Gena with pile a mixture of golden and black, elongate, and dense, forming the beard. Proboscis 1.7–2.5× the length of the body, dorsal and ventral side black. Palpus with first segment significantly longer than that of second segment, second segment much narrower than first segment; colour generally dark brown to black; pile longer on the first segment.

***Thorax*** (Fig. 3B). Scutum dark grey to black; pruinescence mostly silver and brown, with pale grey to silvery pruinescence complete along the lateral sides of the scutum, joining on scutellum; median and paired sublateral vittae evident; medial vitta dark brown, narrow posteriorly, merging with sublateral vittae towards scutellum; paired sublateral vittae dark brown, straight, ending before scutellum; pile mostly black, sparse, shorter than the pile on the posterior of the scutellum; postalar callus with black pile dorsally, ventral side with a tuft of golden pile. Scutellum anterior margin covered by brown pruinescence; posterior margin with a dark, black border; pile on disc of scutellum black, relatively long, sparse compared to scutum; pile along posterior margin elongate, a mixture of black and pale, white or yellow, dense compared to disc of scutellum; with golden and black pile laterally. Pleuron mostly blackish; with silver pruinescence, sparser than on scutum; pile generally a mixture of black and white to yellow, relatively long, of intermediate density; most dense and elongate in two tufts, ventral and anterior to the base of the wing and between postalar callus and posterior spiracle; tuft of pile anterior to wing base directed posteriorly, black pile abutting white to golden pile; with tuft of pile on katatergite directed posteriorly, black and golden; katepimeron with pile absent, or sparse elongate white pile. ***Legs***. Coxae yellow brown to dark brown; with pile mostly black or mostly off-white to golden, elongate, dense. Trochanters mostly blackish, with some yellow-brown colouring; pile short, very sparse. Femora yellow-brown, with dark marking on dorsal side of the distal end present; pile mostly black, mostly short, dense, but with elongate pile dorsally on proximal 1/3–1/2; ventral pile typically longer, sparse; hind femur with short pile more evenly distributed than on fore and mid femora. Tibiae yellow-brown to dark brown; with mostly short pile, most dense on hind tibia. Tarsi red-brown to dark brown, hind tarsi tend to be darker. ***Wings*** (Fig. 5B). Shape relatively slender; broadest just basal to termination of *CuP* on posterior margin; alula broad; costal margin close to straight, without distinct anteriorly curved flexure; *Sc* insertion on *C* aligned with insertion of *M_4_* on posterior margin of wing; *R_1_* inserted closer to *R_2+3_* than to *Sc*; insertions of *Sc* and *R_1_* well separated; short appendix just beyond fork on *R_4+5_* sometimes present; cross vein between *M_1_* and *M_2_* absent; cross vein between *R_4_* and *R_2+3_* absent; *R_1_* relatively straight; *R_4_* shallow bowing upward; *R_5_* shallow bowing upward; *M_1_* and *M_2_* slightly curved upward; cell *cua* open at margin; *CuA* and *CuP* well separated. Dark marking on *R_1_* positioned just basal to humeral cross vein. Membrane with smoky brown infuscation, appearing darker on anterior 1/2–1/3 of wing; posterior region of wing somewhat paler but never hyaline; isolated darker patches indistinct in pale region; the distinction between brown infuscation and hyaline membrane gradual, never striking. Tuft of pile on base of wing white. Haltere with pale brown to yellow stalk; bulb dark brown.

***Abdomen*** (Fig. 3B). Colour of abdomen generally black; T_2_ with posterior margin stout and relatively broad; abdomen tapering abruptly after T_3_. Tergites with silvery pruinescence; membrane between T_1_ and T_2_ with silvery to brown pruinescence; medial brown pruinescent vitta distinct (sometimes indistinct), extending from the posterior margin of T_1_ to terminalia, usually not covering the full length of each tergite. Pile on tergites mostly black, both long and short, of intermediate density; along anterior margins of T_2_ black or white to pale yellow, elongate, sparse; posterolateral pile on T_2_–T_4_ black and white, elongate, dense; T_5_ with pile along lateral margins more evenly distributed than that of T_2_–T_4_. Sternites typically paler than tergites; grey to black; pruinescence silverly with medial brown vitta in some specimens. Sternites with pile a mixture of black and white, mostly long, sparse; pile on S_1_ and S_2_ noticeably longer, white; pile on membrane adjacent to lateral margins of S_2_–S_4_ typically with profuse, decumbent, elongate, pale yellow to golden.

***Genitalia*** (Fig. 7C, D). Hypandrium triangular in shape; broad, tapering abruptly 1/2 way up towards apex; laterally convex, bulging; 1.7× longer than basal width; with apical 1/5 projecting past the top of the gonocoxites. Hypandrium vestiture short, sparse, on the apical 1/2. Gonocoxite apical 1/2 not parallel sided; gonocoxites widest in apical 1/3, narrowing apically; rounded apically. Gonocoxite vestiture on the lateral 1/2, of apical 1/2, mostly short, laterally projecting. Gonostylus narrowed medially; and narrow apical region. Phallus near parallel sided; narrowing apically.

**Female**. Same as male, except for genitalia dimorphism and the following characters: ***Head***. Frons width anterior to ocellar tubercle 0.9× the width above antennal insertions.

##### Geographical distribution.

*Prosoeca marinusi* occurs in a limited area around Nieuwoudtville and towards the Hantamsberg near Calvinia in the Northern Cape Province (Fig. 1).

##### Biology.

*Prosoeca marinusi* can be found on the wing from early August to early November. The individuals found in the Hantamsberg appear to fly slightly later than those from lower lying areas. From specimen labels, *P. marinusi* appears to visit *Lapeirousia jacquinii*, *Lapeirousia oreogena*, *Nemesia cheiranthus*. Visits to *Babiana framesii*, *Lapeirousia montana* (Barraclough, 2018) and *Babiana vanzyliae* (https://www.inaturalist.org/observations/241874048 [accessed 10 February 2025]) have also been reported.

##### Comments.

Individuals from the Hantamsberg tend to be slightly darker in colouration than those from Nieuwoudtville, but the mean genetic differentiation between these populations is 2.06% (range: 1.96%–2.27%).

#### ﻿ Prosoeca peringueyi

Taxon classificationAnimaliaDipteraNemestrinidae

Lichtwardt, 1920

71AC49E3-AC9E-5ECC-8FB3-9F6EB01F0FFB

Figs 1, 2D, 3C, 4C, 5C, 6C, 7E, F

Prosoeca peringueyi Lichtwardt, 1920: 98; Bezzi 1924: 175; Bernardi 1973: 258; Bowden 1980: 375; Barraclough 2006: 49.

##### Type material examined.

***Lectotype***: South Africa: • 1 ♂ Northern Cape, Ookiep, Namaqua Div; R. Lightfoot leg.; Sep. 1990; SAM-DIP-A009013; SAMC. This specimen is hereby designated as lectotype. ***Paralectotype***: South Africa • 1 ♀ Northern Cape, Namaqualand, Klipfontein, Aug. 1890; R. M. Lightfoot; SAM-DIP A009009; SAMC. This specimen is hereby designated as paralectotype.

##### Other material examined.

South Africa: • 1 ♂ 1 ♀, Northern Cape: Van Rhynsdorp; 28 Jul. 1927; Dr. Brauns leg.; *Lapeirousia fissifolia*, [illegible]; NMSA-DIP 49945, NMSA-DIP 049943; NMSA. • 2 ♂♂ 1 ♀ Western Cape: Clanwilliam district: Pakhuis Pass; 950 m; 17–19 Oct.1964; B & P Stuckenberg leg.; NMSA-DIP 052845, NMSA-DIP 76251, NMSA-DIP 76252; NMSA. • 1 ♀ Western Cape: Pakhuis Mts: Pakhuis Farm 2 miles NNE; 14 Sep. 1972; Irwin M.E., Irwin B.J. leg.; NMSA-DIP 054873; NMSA. • 1 ♂ Western Cape: Vanrhynsdorp: Gifberge; Sep. 1911; NMSA-DIP 055007; NMSA. • 1 ♀ Northern Cape: 5 km N of Komaggas; 29°45'S, 17°31'E; 390 m; #85; 24 Aug. 1995; J & A Londt leg.; Rocky slope; Macchia; NMSA-DIP 50895; NMSA. • 5 ♀♀ Western Cape: Clanwilliam; Sep. 1928; Dr. Brauns leg.; NMSA-DIP 76253–76255, NMSA-DIP 76248, NMSA-DIP 76249; NMSA. • 1 ♂ Western Cape: Clanwilliam; Dr. Brauns leg.; Sep. 1928; NMSA-DIP 76250; NMSA. • 2 ♀♀ Western Cape Vanrynsdorp; Jul.-Aug. 1927; G. v. Son leg.; NMSA-DIP 52017, NMSA-DIP 51796; NMSA. • 1 ♂ Western Cape: Clanwilliam: Karroo-berg; G. van Son leg.; 9 Aug. 1927; NMSA-DIP 51950; NMSA. • 1 ♀ Northern Cape: Studers Pass road: NE of Garies; Late Aug.-early Sep.2005; sweep-net; G.B.P. Davies leg.; NMSA-DIP 078933; NMSA. • 1 ♂ 1 ♀ Western Cape: Clanwilliam area: Cederberg mnts: Pakhuis Pass: Kleinkliphuis Farm; G.B.P. Davies leg.; NMSA-DIP 078929, NMSA-DIP 078930; NMSA. • 4 ♂♂ 1 ♀ Northern Cape: Nama Khoi: Naries; 29.701°S, 17.665°E; 5 Aug. 2007; T. van der Niet leg.; NMSA-DIP 219356 –219359, NMSA-DIP 219355; NMSA. • 1 ♀ Western Cape Province: Clanwilliam Dam: E bank; 23 Sep. 1996; F.W., S.K. & R.W. Gess leg.; 96/97/209, on purple fls, *Lapeirousia* sp. Iridaceae; AMGS-ENT 101701; AMGS. • 1 ♂ Western Cape: Vanryansdorp: Gifberg; 1 Sep. 1911; SAM-DIP A009010; SAMC. • 1 ♂ 1 ♀ Western Cape: Clanwilliam: Pakhuis pass; 1 Sep. 1942; South African Museum Expedition leg.; SAM-DIP A009008; SAMC. • 1 ♀ Northern Cape: Namaqualand: Bowesdorp; SA Museum leg.; SAM-DIP A009011; SAMC. • 2 ♀♀ Northern Cape: Namaqualand; 2917 D8; Hester Malan N. R.; M. Struck leg.; 13 Sep. 1986; SAM-DIP A012367, SAM-DIP A009007; SAMC. • 1 ♀ Northern Cape: Soebatsfontein turnoff from N7; 3017 BB; 6 Sep. 1986; K. Steiner leg.; 1428; *Babiana*; SAM-DIP A012381; SAMC. • 1 ♀ Western Cape: Clanwilliam: Ramskop Camp; -32.18, 18.88; K Steiner leg.; 1736; 21 Aug. 1984; *Lapeirousia jacquinii*; SAM-DIP A012382; SAMC. • 2 ♂♂ Northern Cape: 12 km N of Steinkopf; 2917 BB; 4 Aug. 1988; K. Steiner leg.; *Sutera fruticosa*; SAM-DIP A012386; SAMC. • 1 ♀ Western Cape: Clanwilliam: Ramskop; Macpherson leg.; 22 Aug. 1984; *Laperiousia jaquenii*; SAM-DIP A009012; SAMC. • 1 ♂ Western Cape: 10 km W. Algeria, Clanwilliam road, 32.21°S, 19.03°E; 4 Sep.1987; C.D. Eardley leg.; SANC.

##### Diagnosis.

Large-sized (length 13–21 mm), grey body with intricate patterning on the thorax and the abdomen, thorax with two dark black sub-lateral vittae extending to the transverse suture, legs dark brown, proboscis length 1.55 ± 0.02× the length of the body (range of un-extended proboscis length 23–35 mm), wings with smoky brown patterned infuscation on the anterior 1/2 of the wing and cross-vein between *R_4_* & *R_2+3_* complete in specimens from the northern part of the range (north of Calvinia). *Prosoeca peringueyi*, *P. marinusi*, and *P. torquata* can be distinguished from all other species in the clade by their proboscis which is longer than the length of their bodies, in contrast to *P. ora* sp. nov., *P. aquilo* sp. nov., and *P. parva* sp. nov. that have a proboscis shorter than the length of their body. *Prosoeca peringueyi* and *P. torquata* have a dark smoky brown wing patterning that is strikingly distinct from the almost hyaline posterior wing membrane, while *P. marinusi* has a paler brown wing patterning that is never strikingly distinct from the lightly infuscated posterior wing membrane (Fig. 5A–C). *Prosoeca peringueyi* differs most noticeably from *P. torquata* and *P. marinusi*, by the presence of two dark black sublateral vittae on the anterior of the thorax. Additionally, *P. peringueyi* has a generally paler thorax than *P. torquata or P. marinusi*.

##### Redescription.

**Male**. Body length: mean 18.1 mm (*n* = 43); range 13–21 mm. Intertegular width: mean 7.0 mm; range 5–8 mm (*n* = 11). Proboscis length: mean 29.2 mm; range 23–35 mm (*n* = 29). Wing length: mean 19.6 mm; range 18–21 mm (*n* = 19).

***Head***. (Figs 4C, 6C) Ground colour generally red-brown to dark brown. Ocellar tubercle somewhat bulbous and developed, just evident above upper eye margin in profile, with dense silvery pruinescence; width between eyes at the anterior ocellus 3.9–4.2× the length of the anterior ocellus; anterior ocellus separated from posterior ocelli by shallow transverse groove; pile generally long, black. Frons trapezoid; width anterior to ocellar tubercle 0.7× the width above antennal insertions; slightly to moderately swollen between antennal insertions and anterior ocellus; swelling recedes strongly towards eye margin; pruinescence relatively dense, brownish; pile generally absent. Antenna with scape 1.5–2× length of pedicel; first flagellomere subequal to the length of scape + pedicel; style longer than scape + pedicel + flagellomere 1; ground colour transitioning to a darker hue, on the proximal side of the first flagellomere, style darker than remainder of antenna; scape, pedicel and flagellomere 1 with irregular silver to brown pruinescence; pedicel with both elongate and short pile, black and white pile, flagellomere 1 with short black pile basally on the dorsal side. Facial area bulbous in profile, with horizontal groove present; sublaterally irregular yellow-brown; with silver to brown pruinescence, but pruinescence largely absent from medial section of face; pile mostly white, elongate, sparse, more dense than on frons. Gena with pile a mixture of black and white to pale yellow, elongate, and dense, forming the beard. Proboscis 1.2–1.7× the length of the body, black with dorsal part of basal 1/3–1/2 brown. Palpus with first segment significantly longer than that of second segment, second segment much narrower than first segment; colour generally dark brown to black; pile longer on the first segment.

***Thorax*** (Fig. 3C). Scutum dark grey to black; pruinescence mostly silver and brown, with pale grey to silvery pruinescence complete along the lateral sides of the scutum, joining on scutellum; median and paired sublateral vittae evident; medial vitta narrow anteriorly, widening towards scutellum; paired sublateral vittae distinct black, straight, ending at transverse suture; pruinescence forming irregular pattern; pile mostly black, sparse, shorter than the pile on the posterior of the scutellum; pile on postpronotal lobe and anterodorsal region of scutum white, sparse; postalar callus with black pile dorsally, ventral side with a tuft of white pile. Scutellum with distinct central dot, or central diamond shaped marking; anterior margin covered by silver to brown pruinescence; posterior margin with a dark, black border; pile on disc of scutellum black, relatively long, sparse compared to scutum; pile along posterior margin elongate, mostly black, dense compared to disc of scutellum; with some pale yellow to golden pile laterally (sometimes closer to white). Pleuron mostly blackish; with silver pruinescence, sparser than on scutum; pile generally a mixture of black and white to yellow, relatively long, of intermediate density; most dense and elongate in two tufts, ventral and anterior to the base of the wing and between postalar callus and posterior spiracle; tuft of pile anterior to wing base directed posteriorly, mostly white to golden with some black pile; tuft of pile on katatergite directed posteriorly, black and golden; katepimeron with pile absent, or sparse elongate white pile. ***Legs***. Coxae red-brown to dark brown; with pile mostly off-white to golden, elongate, dense. Trochanters mostly blackish, with some yellow-brown colouring; pile short, very sparse. Femora red-brown to dark brown, with dark marking on dorsal side of the distal end present; pile mostly black, mostly short, dense, but with elongate pile dorsally on proximal 1/3 –1/2; ventral pile typically longer, sparse; hind femur with short pile more evenly distributed than on fore and mid femora. Tibiae red-brown to black; with mostly short pile, most dense on hind tibia. Tarsi red-brown to dark brown, hind tarsi tend to be darker. ***Wings*** (Fig. 5C). Shape relatively slender; broadest just basal to termination of *CuP* on posterior margin; alula broad; costal margin close to straight, without distinct anteriorly curved flexure; *Sc* termination on *C* aligned with termination of *M_4_* on posterior margin of wing; *R_1_* terminated closer to *R_2+3_* than to *Sc*; insertions of *Sc* and *R_1_* well separated; cross vein between *M_1_* and *M_2_* absent; cross vein between *R_4_* and *R_2+3_* just beyond fork of *R_4_* and *R_5_* present, or absent; *R_1_* relatively straight; *R_4_* deep bowing upward; *R_5_* shallow bowing upward; *M_1_* and *M_2_* slightly curved upward; cell *cua* open at margin; *CuA* and *CuP* well separated. Dark marking on *R_1_* positioned just basal to humeral cross vein; membrane with smoky brown infuscation; appearing darker on anterior 1/2–1/3 of wing; posterior region of wing almost hyaline; isolated darker patches distinct in hyaline region; the distinction between brown infuscation and hyaline membrane sharply delineated. Tuft of pile on base of wing white. Haltere with pale brown to yellow stalk; bulb dark brown.

***Abdomen*** (Fig. 3C). Colour of abdomen generally red-brown; T_2_ with posterior margin stout and relatively broad; abdomen tapering abruptly after T_3_. Tergites with silvery pruinescence; membrane between T_1_ and T_2_ with silvery to brown pruinescence; medial brown pruinescent vitta distinct, extending from the posterior margin of T_1_ to terminalia, usually not covering the full length of each tergite; paired sublateral markings of indistinct shape, on T_2_–T_4_. Pile on tergites mostly black, both long and short, of intermediate density; along anterior margins of T_2_ white to pale yellow, elongate, sparse; posterolateral pile on T_2_–T_4_ black, elongate, dense; T_5_ with pile along lateral margins more evenly distributed than that of T_2_–T_4_. Sternites typically paler than tergites; pale reddish brown; pruinescence silver with medial brown vittae, relatively sparse. Sternites with pile a mixture of black and white, mostly short, sparse; pile on S_1_–S_3_ intermixed, long, white; membrane adjacent to lateral margins of S_2_–S_4_ typically with profuse, decumbent, elongate, white pile.

***Genitalia*** (Fig. 7E, F). Hypandrium triangular in shape; relatively narrow; laterally straight sided; 1.8× longer than basal width; with apical 1/5 projecting past the top of the gonocoxites. Hypandrium vestiture long, dense, on the apical 1/3. Gonocoxite apical 1/2 parallel sided; gonocoxites widest in basal 1/2, narrowing apically; rounded apically. Gonocoxite vestiture on the lateral 1/2, of apical 1/3, mostly short, laterally projecting. Gonostylus with parallel sides; and narrow apical region. Phallus near parallel sided; narrowing apically.

**Female**. Same as male, except for genitalia dimorphism and the following characters: ***Head***. Frons width anterior to ocellar tubercle 0.8× the width above antennal insertions.

##### Geographical distribution.

*Prosoeca peringueyi* is the most widespread species in this clade. It occurs from Khuboes near the Namibia/South Africa border in the north to Piekenierskloof pass in the Western Cape Province in the south (pers. obs., A.G. Ellis), with a distribution gap within the Knersvlakte (Fig. 1).

##### Biology.

The *P. peringueyi* complex is known to visit many plant species (Manning and Goldblatt 1996; Manning and Goldblatt 2000; Pauw et al. 2020) in both Fynbos and Succulent Karoo biomes. It is however, not precisely known for all plant species whether *P. peringueyi* and *P. torquata* are pollinators of the same suite of plants or if they partition these floral resources. *Prosoeca peringueyi* is known with some certainty, from specimen labels, to visit *Lapeirousia pyramidalis*, *Pelargonium magenteum*, *Lapeirousia silenoides*, *Lapeirousia jacquinii*, *Pelargonium incrassatum*, and *Sutera fruticosa*.

##### Comments.

*Prosoeca peringueyi* has a degree of genetic differentiation between the populations north and south of the Knersvlakte (mean 6.27%). This genetic disjunction is supported by a difference in wing venation, with the cross vein between *R_4_* and *R_2+3_* just beyond fork of *R_4_* and *R_5_* present north of the Knersvlakte and absent in the south. As the wing vein character is not functional and the genetic difference below the usual threshold for species delineation, we consider this to be a single species.

#### ﻿ Prosoeca aquilo sp. nov.

Taxon classificationAnimaliaDipteraNemestrinidae

4ED31217-87E4-5B1D-9E0C-B2DCEAFB305A

https://zoobank.org/59D99077-ECC7-48A7-AE0A-C9159DD3BE90

**Figure 8.**
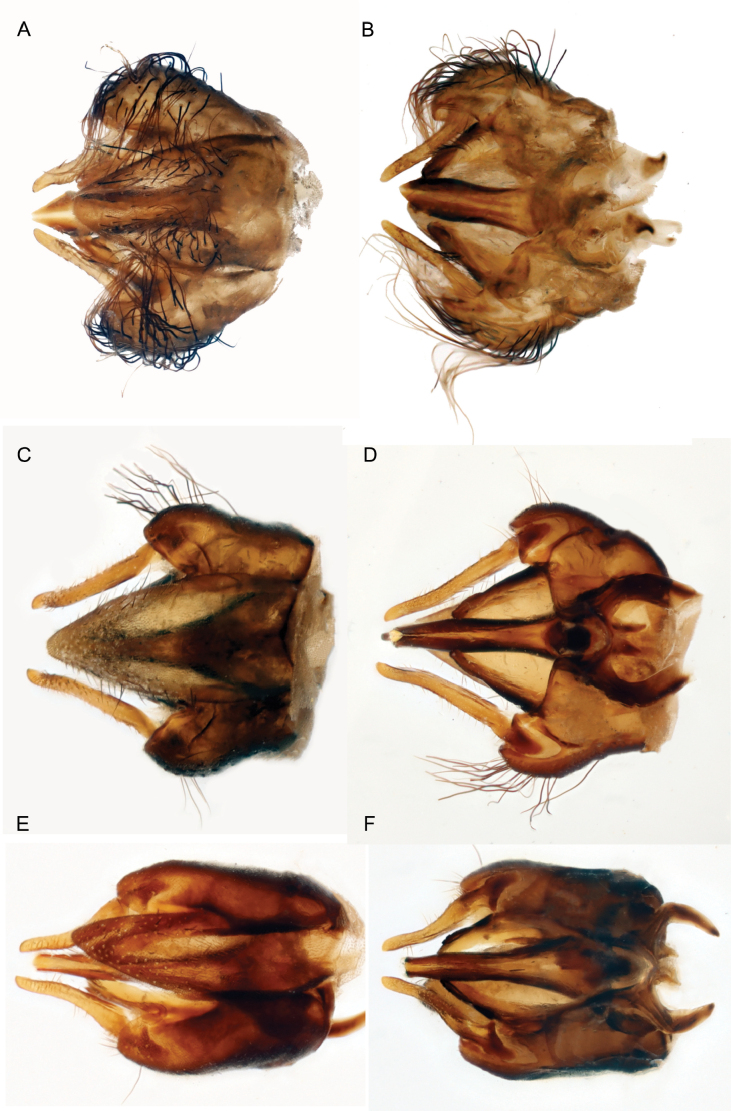
Genitalia of A, B. *P. aquilo* sp. nov., C, D. *P. ora* sp. nov., E, F. *P. parva* sp. nov. A, C, E. dorsal view; B, D, F. ventral view.

Figs 1, 3D, 4D, 5D, 6D, 8A, B

##### Type material.

***Holotype***: South Africa: • 1 ♂ Northern Cape: Kamieskroon: S. side Rooiberg Mts, high altitude fynbos, wetland stream; 30°26.276'S, 18°05.140'E; 1372 m; 23 Aug. /2005; J. Coville & A. Roberts leg.; NMSA-DIP 79006; NMSA. ***Paratypes***: South Africa: • 2 ♂♂ Northern Cape: Kamieskroon: S. side Rooiberg Mts, high altitude fynbos, wetland stream; 30°26.276'S, 18°05.140'E; 1372 m; 23 Aug. 2005; J. Coville & A. Roberts leg.; NMSA-DIP 79002, NMSA-DIP 79005; NMSA. • 1 ♂ Northern Cape: Kamieskroon: S. side Rooiberg Mts, high altitude fynbos, wetland stream; 30°26.276'S, 18°05.140'E; 1372 m; 23 Aug. 2005; J. Colville & A. Roberts leg.; RMCA-ENT 000056700; RMCA.

##### Diagnosis.

Relatively small species (length 11–12 mm) with a conspicuous grey border on the thorax and scutellum, abdomen black with posterior grey fascia interrupted by brown medial vitta, femora generally darker than tibiae and tarsi, proboscis length 0.8 ± 0.02× the length of the body (range of un-extended proboscis length 8–10 mm), and wings with smoky brown infuscation on the anterior margin. *Prosoeca aquilo* sp. nov. can be distinguished from all others in the clade by the combination of its small size, the wings with smoky brown infuscation anteriorly and a paler posterior region and slight but distinct flexure on the anterior of the wing in males (Fig. 5). *Prosoeca aquilo* sp. nov. differs from *P. parva* sp. nov. by having distinct infuscation on the anterior of the wing while *P. parva* sp. nov. has very little infuscation and wings are almost entirely hyaline (Fig. 5D, F). *Prosoeca ora* sp. nov. has an abdominal pattern closely resembling that of *P. aquilo* sp. nov.; however, *P. aquilo* sp. nov. has grey pruinescence restricted to the posterior border of the tergites, not encircling the brown medial vitta, while *P. ora* sp. nov. has more extensive grey pruinescence medially (Fig. 3D, E). Furthermore, the dense black pile laterally on the frons separates *P. aquilo* sp. nov. from *P. ora* sp. nov., which usually has white pile on these areas (Fig. 6D, E). *Prosoeca aquilo* sp. nov. differs most notably from *P. marinusi*, *P. peringueyi*, and *P. torquata* by having a small body size and a proboscis shorter than the length of its body.

##### Description.

**Male**. Body length: mean 11.6 mm; range 11–12 mm (*n* = 4). Intertegular width: mean 4.4 mm; range 4–5 mm (*n* = 4). Proboscis length: mean 9 mm; range 8–10 mm (*n* = 4). Wing length: mean 13.2 mm; range 12–14 mm (*n* = 4).

***Head***. (Figs 4D, 6D) Ground colour generally grey to black. Ocellar tubercle somewhat bulbous and developed, just evident above upper eye margin in profile, with dense silvery pruinescence; width between eyes at the anterior ocellus 3–3.5× the length of the anterior ocellus; anterior ocellus separated from posterior ocelli by shallow transverse groove; pile generally long, black. Frons trapezoid; width anterior to ocellar tubercle 0.6× the width above antennal insertions; slightly to moderately swollen between antennal insertions and anterior ocellus; swelling recedes strongly towards eye margin; pruinescence relatively dense, silver to brown; pile generally pale with black pile dorsolaterally, dense along lateral margins towards antennal insertions, but sparse medially. Antenna with scape 1–1.3× length of pedicel; first flagellomere subequal to the length of scape + pedicel; style longer than scape + pedicel + flagellomere 1; ground colour dark brown to black, style darker than remainder of antenna; scape, pedicel and flagellomere 1 with irregular silver to brown pruinescence; pedicel with mostly elongate pile, black pile, flagellomere 1 with short black pile basally on the dorsal side. Facial area bulbous in profile, with horizontal groove present; with silver to brown pruinescence, evenly distributed across face; pile mostly white (sometimes with black pile interspersed), elongate, sparse, similar to that of frons. Gena with pile a mixture of black and white to pale yellow, elongate, and dense, forming the beard. Proboscis 0.7–0.9× the length of the body, dorsal and ventral side black. Palpus with first segment significantly longer than that of second segment, second segment much narrower than first segment; colour generally dark brown to black; pile long on both segments.

***Thorax*** (Fig. 3D). Scutum dark grey to black; pruinescence mostly brown, with pale grey to silvery pruinescence complete along the lateral sides of the scutum, joining on scutellum; median and paired sublateral vittae absent; pile mostly black (sometimes with a few golden pile interspersed), sparse, shorter than the pile on the posterior of the scutellum; postalar callus with black pile dorsally, ventral side with a tuft of golden pile. Scutellum anterior margin covered by brown pruinescence; posterior margin with a dark, black border; pile on disc of scutellum black, relatively long, of similar density to that on the scutum; pile along posterior margin elongate, a mixture of black and pale, white or yellow, same density as on disc of scutellum; with some pale yellow to golden pile laterally. Pleuron mostly blackish; with silver pruinescence, sparser than on scutum; pile generally a mixture of black and white to yellow, relatively long, of intermediate density; most dense and elongate in two tufts, ventral and anterior to the base of the wing and between postalar callus and posterior spiracle; tuft of pile anterior to wing base directed posteriorly, mostly white to golden with some black pile; tuft of pile on katatergite directed posteriorly, black and golden; katepimeron with pile absent. ***Legs***. Coxae dark brown to black; with pile mostly off-white to golden, elongate, dense. Trochanters mostly blackish, with some yellow-brown colouring; pile short, very sparse. Femora yellow-brown (sometimes very dark to black), with dark marking on dorsal side of the distal end present; pile mostly black, mixed long and short, dense, but with elongate pile dorsally on proximal 1/3 to 1/2; ventral pile typically longer, sparse; hind femur with short pile more evenly distributed than on fore and mid femora. Tibiae yellow-brown to dark brown; with dense, short, dark pile and sparse, elongate pile (short pile may appear more golden on hind tibia), most dense on hind tibia. Tarsi red-brown to dark brown, hind tarsi tend to be darker. ***Wings*** (Fig. 5D). Shape relatively slender; broadest distal to termination of *M_4_* on posterior margin; alula broad; costal margin with slight but distinct anterior curved flexure; *Sc* termination on *C* aligned with termination of *M_4_* on posterior margin of wing; *R_1_* termination closer to *R_2+3_* than to *Sc*; termination of *Sc* and *R_1_* well separated; short appendix just beyond fork on *R_4+5_* always absent; cross vein between *M_1_* and *M_2_* absent; cross vein just beyond fork between *R_4_* and *R_2+3_* absent; *R_1_* slightly curved upward; *R_4_* deep bowing upward; *R_5_* deeply bowing upward; *M_1_* and *M_2_* slightly curved upward; cell *cua* open at margin; *CuA* and *CuP* well separated. Dark marking on *R_1_* positioned just basal to humeral cross vein; membrane with smoky brown infuscation; infuscated on anterior 1/2–1/3 of wing; posterior region of wing somewhat paler but never hyaline; isolated darker patches distinct in pale region; the distinction between brown infuscation and pale brown membrane clearly delineated. Tuft of pile on base of wing white. Haltere with pale brown to yellow stalk; bulb dark brown.

***Abdomen*** (Fig. 3D). Colour of abdomen generally black; T_2_ with posterior margin stout and relatively broad; abdomen tapering abruptly after T_3_. Tergites with silvery pruinescence; membrane between T_1_ and T_2_ with silvery to brown pruinescence; medial brown pruinescent vitta distinct, extending from the posterior margin of T_1_ to terminalia, usually not covering the full length of each tergite; grey pruinescence surrounding brown medial vitta, on T_2_–T_5_; posterior margin of T_2_–T_4_ with contrasting pruinescent border. Pile on tergites mostly black, both long and short, of intermediate density; along anterior margins of T_2_ white to pale yellow (with some dark pile medially), elongate, of intermediate density; posterolateral pile on T_2_–T_4_ black, elongate, dense; T_5_ with pile along lateral margins more evenly distributed than that of T_2_–T_4_. Sternites typically paler than tergites; grey to black; pruinescence silvery, dense. Sternites with pile mostly white, mostly long, sparse; pile on S_4_–S_5_ black; pile on membrane adjacent to lateral margins of S_2_–S_4_ typically with profuse, decumbent, elongate, white pile.

***Genitalia*** (Fig. 8A, B). Hypandrium triangular in shape; broad, tapering gradually towards the apex; laterally convex; 1.7× longer than basal width; with apex projecting only slightly past the top of the gonocoxites. Hypandrium vestiture long, sparse, on the apical 2/3. Gonocoxite apical 1/2 not parallel sided; gonocoxites widest in apical 1/3, narrowing apically; rounded apically. Gonocoxite vestiture on the lateral 2/3, of apical 2/3, long, laterally projecting. Gonostylus with parallel sides; and narrow apical region. Phallus near parallel sided; narrowing apically.

**Female**. Unknown

##### Geographical distribution.

Only known from a single locality in the Kamiesberg mountains near Leliefontein in the Northern Cape of South Africa (Fig. 1).

##### Biology.

Found visiting yellow *Oxalis* in marshy wetland, high-altitude Fynbos. This species has only been recorded on the wing in late August but is likely to be on the wing through most of the spring season.

##### Etymology.

From the Latin *aquilo* = Roman god of the north winds; referring to its current known northerly distribution in the Kamiesberg. To be treated as a noun in apposition.

#### ﻿ Prosoeca ora sp. nov.

Taxon classificationAnimaliaDipteraNemestrinidae

38EAD2FB-03B0-5E85-8C87-D9CED1CE7E39

https://zoobank.org/403CCA4C-9ACF-4C98-8C41-501046F356E4

Figs 1, 2B, 2C, 3E, 4E, 5E, 6E, 8C, D

##### Type material.

***Holotype***: South Africa: • 1 ♂, Northern Cape: Hantam Botanical Garden; 27 Aug. 2017; T. van der Niet leg.; NMSA-DIP 219345; NMSA. ***Paratypes***: South Africa: • 2 ♀♀ Northern Cape: Nieuwoudtville; 26 Aug. 1999; P. Goldblatt & J. Manning leg.; visiting *Babiana vanzyliae* (Iridaceae); NMSA-DIP 79017, NMSA-DIP 79018; NMSA. • 1 ♂ Northern Cape: Sutherland: Komsberg; J. Manning leg.; NMSA-DIP 54396; NMSA. • 1 ♀ Northern Cape: Calvinia: Hantamsberg; 3 Sep.1994; 29; on *Pteronia incana*; NMSA-DIP 54393, [handwriting of Dr. J. Manning]; NMSA. • 1 ♂ Western Cape: Middelpos: Gannaka Pass; 1 Jul.1993; NMSA-DIP 76672, [handwriting of Dr. J. Manning]; NMSA. • 1 ♂ 4 ♀♀ Northern Cape: Sutherland: Ouberg; 24–25 Sep. 2022; visiting *Dimorphotheca cuneata*; Allan Ellis leg.; NMSA-DIP 219360–NMSA-DIP 219364; NMSA. • 1 ♀ Northern Cape: Smoushoogte Pass: De Plaat; 32.81363°S, 20.70072°E; 17 Oct. 2008; Anton Pauw leg.; AP414; NMSA-DIP 221842; NMSA. • 1 ♀ Northern Cape: Nieuwoudtville: Greylon Renosterveld; 03 Oct.2012; Anton Pauw leg.; AP674; visiting *Hesperantha cucullata*; NMSA-DIP 221843; NMSA. • 1 ♀ Northern Cape: Nieuwoudtville: Glenlyon; Renosterveld; 3 Sep. 2012; Anton Pauw leg.; AP675; RMCA-ENT 000056701; RMCA. • 1 ♂ Northern Cape: Nieuwoudtville: Glenlyon; Renosterveld; 3 Sep. 2012; Anton Pauw leg.; AP676; RMCA-ENT 000056702; RMCA.

##### Other material examined.

South Africa: • 1 ♀ Western Cape: Maitjiesfontein Koppie: E. Soetwater; 07 Sep. 2019; Allan Ellis leg.; NMSA-DIP 219351; NMSA. • 2 ♂♂ Northern Cape: Hantam Botanical Garden; 27 Aug. 2017; T. van der Niet leg.; NMSA-DIP 219344, NMSA-DIP 219346; NMSA. • 3 ♂♂ Northern Cape: Hantamsberg; -31.39563°S, 19.78919°E; 18 Sep. 18; Florent Grenier leg.; NMSA-DIP 219348–NMSA-DIP 219350; NMSA. • 1 ♂ Western Cape: 7 km N of Wuppertal: North slope; 32°14'S, 19°10'E; 780 m; 31 Aug. 1995; J & A Londt leg.; Flowers; NMSA-DIP 50966; NMSA. • 1 ♀ Western Cape: 7 km N of Wuppertal: North slope; 32°14'S, 19°10'E; 780 m; 31 Aug. 1995; J & A Londt leg.; Flowers; NMSA-DIP 76690; NMSA. • 1 ♂ 1 ♀ National Road 22 m from Touws River; 3 Oct. 1964; B. & P. Stuckenberg leg.; NMSA-DIP 52836, NMSA-DIP 78337; NMSA. • 1 ♂ Nothern Cape: Middelpos: Gannaka Pass; 1 Jul. 1993; NMSA-DIP 51673 [handwriting of Dr. J. Manning] • NMSA. 2 ♂♂ Northern Cape: Smoushoogte Pass: De Plaat; 32.81363°S, 20.70072°E; 17 Oct. 2008; Anton Pauw leg.; AP414; visiting yellow annual *Pseudoselago*; NMSA-DIP 221840- NMSA-DIP 221841; NMSA. • 1 ♀ Western Cape: Heuningvlei; 32.20636°S, 19.07513°E; #SIM1207C; SI Morita leg.; *Prosoeca peringueyi*; CDFA-ENT; CDFA. • 1 ♀ Northern Cape: Nieuwoudtville: Trekpad; 3 Sep. 2012; Anton Pauw leg.; AP673 RMCA-ENT 000056703; RMCA. • 1 ♂ Northern Cape: Sutherland: Smoushoogte; 32°48'49.1"S, 20°42'02.6"E; 7 Oct. 2008; Anton Pauw leg.; AP415; RMCA-ENT 000056704; RMCA.

##### Diagnosis.

Medium-sized (length 10–17 mm), thorax dark, scutellum and thorax with conspicuous grey border, abdomen black but tergites interrupted medially by a pale grey band, often flaring out posteriorly, and a dark brown median vitta, femora dorsally darker than tibia and tarsi, proboscis length 0.7 ± 0.02× the length of the body (range of un-extended proboscis length 6–11 mm). *Prosoeca ora* sp. nov. can be distinguished from all other species in the clade by the distinct grey, brown and black patterning on the abdomen (Figs 2B, C, 3E). Unlike *P. parva* sp. nov. which has hyaline wings, the wings of *P. ora* sp. nov. are infuscated on the anterior ¼ with a relatively straight costal margin in both sexes, whereas *P. aquilo* sp. nov. has a slight flexure in the costal margin of males (Fig. 5D–F). *Prosoeca ora* sp. nov. has a proboscis that is shorter than the length of its own body, unlike *P. marinusi*, *P. torquata*, and *P. peringueyi*.

##### Description.

**Male**. Body length: mean 13.8 mm; range 10–17 mm (*n* = 19). Intertegular width: mean 6.5 mm; range 4–8 mm (*n* = 9). Proboscis length: 9.7 mm; range 6–11 mm (*n* = 18). Wing length: mean 15.4 mm; range 11–18 mm (*n* = 19).

***Head***. (Figs 4E, 6E) Ground colour generally grey to black. Ocellar tubercle somewhat bulbous and developed, just evident above upper eye margin in profile, with dense silvery pruinescence; width between eyes at the anterior ocellus 2.5–3.5× the length of the anterior ocellus; anterior ocellus separated from posterior ocelli by shallow transverse groove; pile generally long, black. Frons trapezoid; width anterior to ocellar tubercle 0.5–0.6× the width above antennal insertions; slightly to moderately swollen between antennal insertions and anterior ocellus; swelling recedes strongly towards eye margin; pruinescence relatively dense, silver to brown; pile mostly white (sometimes with black pile dorsally), usually dense on entire frons (sometimes sparse). Antenna with scape 1–1.5× length of pedicel; first flagellomere shorter than the length of scape + pedicel; style shorter than scape + pedicel + flagellomere 1; ground colour dark brown to black, style darker than remainder of antenna; scape, pedicel and flagellomere 1 with irregular silver to brown pruinescence; pedicel with mostly elongate pile, black pile, flagellomere 1 with short black pile basally on the dorsal side. Facial area bulbous in profile, with horizontal groove present; with silver to brown pruinescence, evenly distributed across face; pile mostly white, elongate, usually dense (sometimes sparse), similar to that of frons. Gena with pile mostly off-white to golden (sometimes with some black pile), elongate and dense, forming the beard. Proboscis 0.6–0.8× the length of the body, dorsal and ventral side black. Palpus with first segment significantly longer than that of second segment, second segment much narrower than first segment; colour generally dark brown to black; pile long on both segments.

***Thorax*** (Fig. 3E). Scutum dark grey to black; pruinescence mostly brown, with pale grey to silvery pruinescence complete along the lateral sides of the scutum, joining on scutellum; median and paired sublateral vittae absent; pile mixture of black and pale to golden, sparse, shorter than the pile on the posterior of the scutellum; postalar callus with black pile dorsally, ventral side with a tuft of golden pile. Scutellum anterior margin covered by brown pruinescence; posterior margin with a dark, black border; pile on disc of scutellum golden or black, relatively long, sparse compared to scutum; pile along posterior margin elongate, a mixture of black and pale, white or yellow, same density as on disc of scutellum; with some pale yellow to golden pile laterally. Pleuron mostly blackish; with silver pruinescence, sparser than on scutum; pile generally a mixture of black and white to yellow, relatively long, of intermediate density; most dense and elongate in two tufts, ventral and anterior to the base of the wing and between postalar callus and posterior spiracle; tuft of pile anterior to wing base directed posteriorly, mostly white to golden with some black pile; tuft of pile on katatergite directed posteriorly, black and golden (sometimes mostly pale yellow to golden); katepimeron with sparse elongate white pile. ***Legs***. Coxae dark brown to black; with pile mostly off-white to golden, elongate, dense. Trochanters mostly blackish, with some yellow-brown colouring; pile short, very sparse. Femora red-brown to dark brown (sometimes more yellow-brown), with dark marking on dorsal side of the distal end present; pile short golden with long black and white interspersed, mostly short, dense, but with elongate pile dorsally on proximal 1/3–1/2; ventral pile typically longer, sparse; hind femur with short pile more evenly distributed than on fore and mid femora. Tibiae red-brown to black (sometimes closer to yellow-brown); with dense, short, pale pile and sparse, darker, elongate pile, most dense on hind tibia. Tarsi red-brown to dark brown, hind tarsi tend to be darker. ***Wings*** (Fig. 5E). Shape relatively slender; broadest just basal to termination of *CuP* on posterior margin; alula broad; costal margin close to straight, without distinct anteriorly curved flexure; *Sc* termination on *C* aligned with termination of *M_4_* on posterior margin of wing; *R_1_* terminated closer to *R_2+3_* than to *Sc*; termination of *Sc* and *R_1_* well separated; short appendix just beyond fork on *R_4+5_* always absent; cross vein between *M_1_* and *M_2_* absent; cross vein just beyond fork between *R_4_* and *R_2+3_* absent; *R_1_* relatively straight; *R_4_* deep bowing upward; *R_5_* deeply bowing upward; *M_1_* and *M_2_* gently bowing upward; cell *cua* open at margin; *CuA* and *CuP* well separated. Dark marking on *R_1_* positioned just basal to humeral cross vein; membrane with smoky brown infuscation; appearing darker on anterior 1/2–1/3 of wing; posterior region of wing somewhat paler but never hyaline; isolated darker patches distinct in pale region; the distinction between brown infuscation and pale membrane gradual, never striking. Tuft of pile on base of wing white. Haltere with pale brown to yellow stalk; bulb dark brown.

***Abdomen*** (Fig. 3E). Colour of abdomen generally black; T_2_ with posterior margin stout and relatively broad; abdomen tapering abruptly after T_3_. Tergites with silvery pruinescence; membrane between T_1_ and T_2_ with silvery to brown pruinescence; medial brown pruinescent vitta distinct, extending from the posterior margin of T_1_ to terminalia, usually not covering the full length of each tergite; grey pruinescence surrounding medial vitta, on T_2_–T_5_; posterior margin of T_2_–T_4_ with contrasting pruinescent border. Pile on tergites mostly black, both long and short, of intermediate density; along anterior margins of T_2_ white to pale yellow (sometimes more golden), elongate, dense; posterolateral pile on T_2_–T_4_ black and white, elongate, dense; T_5_ with pile along lateral margins more evenly distributed than that of T_2_–T_4_. Sternites typically paler than tergites; grey to black; pruinescence silvery, relatively sparse (sometimes with relatively dense golden pruinescence). Sternites with pile off-white to golden, mostly long, dense; pile on membrane adjacent to lateral margins of S_2_–S_4_ typically with profuse, decumbent, elongate, pale yellow to golden.

***Genitalia*** (Fig. 8C, D). Hypandrium triangular in shape; broad, tapering gradually towards the apex; laterally convex; 1.9× longer than basal width; with apical 1/3 projecting past the top of the gonocoxites. Hypandrium vestiture short, sparse, on the apical 1/2. Gonocoxite apical 1/2 not parallel sided; gonocoxites widest in apical 1/3, narrowing apically; rounded apically. Gonocoxite vestiture on the lateral 1/2, of apical 2/3, long, laterally projecting. Gonostylus with parallel sides and globular apical section. Phallus near parallel sided; narrowing apically.

**Female**. Same as male, except for genitalia dimorphism and the following characters: ***Head***. Ocellar tubercle width between eyes at the anterior ocellus 3.4–4.5× the length of the anterior ocellus. Frons width anterior to ocellar tubercle 0.6–0.7× the width above antennal insertions. Facial area with horizontal groove present (less pronounced than in males). ***Legs***. Femora dark brown to black (sometimes closer to red-brown).

##### Geographical distribution.

*Prosoeca ora* sp. nov. has been recorded from Nieuwoudtville in the Northern Cape to Matjiesfontein and Touws River in the Western Cape (Fig. 1).

##### Biology.

*Prosoeca ora* sp. nov. is on the wing from early August to mid-October in the winter-rainfall region of the Western and Northern Cape provinces of South Africa. This species has been recorded visiting *Pteronia incana*, *Dimorphotheca cuneata*, *Babiana vanzyliae*, *Hesperantha cucullata* and yellow *Pseudoselago* (Scrophulariaceae). Additionally, based on the representative specimen accessioned into NMSA from Goldblatt and Manning (2007), we note that *Prosoeca ora* sp. nov. is likely the pollinator of *Romulea syringodeoflora*.

##### Etymology.

From the Latin *ora* = edge or rim; referring to the characteristic grey pruinescence on the dark thorax forming a distinct border. To be treated as a noun in apposition.

##### Comment.

The single female specimen from Soetwater (NMSA-DIP 219351, NMSA) shows significant genetic differentiation from other *P. ora* sp. nov. specimens but resembles this species morphologically. This specimen is placed in *P. ora* sp. nov. until further sampling can resolve its relationship within this clade.

#### ﻿ Prosoeca parva sp. nov.

Taxon classificationAnimaliaDipteraNemestrinidae

D1A60800-C15D-5E3D-A470-7CAC3442E8E8

https://zoobank.org/1857A992-E394-483B-B63F-6C3AFE2F19F1

Figs 1, 3F, 4F, 5F, 6F, 8E, F

##### Type material.

***Holotype***: South Africa: • 1 ♂ Northern Cape, Calvinia: Hantamsberg; 3 Sep. 1994; 30; *Felicia*; NMSA-DIP 76671; NMSA. ***Paratype***: South Africa: • 1 ♀ Northern Cape: Calvinia: Hantamsberg; 3 Sep. 1994; 30; *Felicia*; NMSA-DIP 76670; NMSA. • 1 ♀ Northern Cape: Hantamsberg; 31.39563°S, 19.78919°E; 15 Sep. 2018; F. Grenier leg.; #FG46; RMCA-ENT 000056705; RMCA. • 1 ♂, Northern Cape: Hantamsberg; 31.39563°S, 19.78919°E; 15 Sep. 2018; F. Grenier leg.; #FG48; RMCA-ENT 000056706; RMCA.

##### Diagnosis.

Relatively small species (length 8–11 mm), thorax dark without distinct central markings, thorax and scutellum with a conspicuous grey border, abdomen uniformly black, proboscis length 0.57 ± 0.04× the length of the body (range of un-extended proboscis length 4 mm–6 mm), femora very dark with pale red-brown tibia and tarsi and conspicuously short antennal style. *Prosoeca parva* sp. nov. can be distinguished from all other species in the clade by the apparent lack of patterning on the abdomen (Fig. 3F), largely hyaline wings (Fig. 5F) and the dark femora which contrast the paler tibia and tarsi. *Prosoeca parva* sp. nov. is notably smaller than *P. marinusi*, *P. peringueyi*, and *P. torquata* with a proboscis shorter than the length of its body, usually not reaching past the hind legs when folded beneath body.

##### Description.

**Male**. Body length: mean 9.8 mm; range 8–11 mm (*n* = 4). Intertegular width: mean 3.8 mm; range 3–4 mm (*n* = 4). Proboscis length: mean 5.6 mm; range 4–6 mm (*n* = 4). Wing length: mean 11.2 mm; range 11–12 mm (*n* = 4).

***Head***. (Figs 4F, 6F) Ground colour generally grey to black. Ocellar tubercle somewhat bulbous and developed, just evident above upper eye margin in profile, with dense silvery pruinescence; width between eyes at the anterior ocellus 3.8× the length of the anterior ocellus; anterior ocellus separated from posterior ocelli by shallow transverse groove; pile generally long, black. Frons trapezoid; width anterior to ocellar tubercle 0.7× the width above antennal insertions; slightly to moderately swollen between antennal insertions and anterior ocellus; swelling recedes strongly towards eye margin; pruinescence relatively dense, silver to brown; pile black and white, dense on entire frons. Antenna with scape 1.5–2× the length of pedicel; first flagellomere shorter than the length of scape + pedicel; style shorter than scape + pedicel + flagellomere 1; ground colour dark brown to black, style darker than remainder of antenna; scape, pedicel and flagellomere 1 with irregular silver to brown pruinescence; pedicel with mostly elongate pile, black pile, flagellomere 1 with short black pile basally on the dorsal side. Facial area bulbous in profile, with horizontal groove present; with silver to brown pruinescence, evenly distributed across face; pile mostly white, elongate, dense, similar to that of frons. Gena with pile a mixture of black and white to pale yellow, elongate, and dense, forming the beard. Proboscis 0.5–0.7× the length of the body, dorsal and ventral side black. Palpus with first segment significantly longer than that of second segment, second segment much narrower than first segment; colour generally dark brown to black; pile long on both segments.

***Thorax*** (Fig. 3F). Scutum dark grey to black; pruinescence mostly brown, with pale grey to silvery pruinescence complete along the lateral sides of the scutum, joining on scutellum; median and paired sublateral vittae absent; pile mixture of black and pale to golden, sparse, shorter than the pile on the posterior of the scutellum; postalar callus with black pile dorsally, ventral side with a tuft of golden pile. Scutellum anterior margin covered by brown pruinescence; posterior margin with a dark, black border; pile on disc of scutellum golden or black, relatively long, of similar density to that on the scutum; pile along posterior margin elongate, a mixture of black and pale, white or yellow, same density as on disc of scutellum; with some pale yellow to golden pile laterally. Pleuron mostly blackish; with silver pruinescence, sparser than on scutum; pile generally a mixture of black and white to yellow, relatively long, of intermediate density; most dense and elongate in two tufts, ventral and anterior to the base of the wing and between postalar callus and posterior spiracle; tuft of pile anterior to wing base directed posteriorly, mostly white to golden with some black pile; tuft of pile on katatergite directed posteriorly, black and golden; katepimeron with pile absent. ***Legs***. Coxae dark brown to black; with pile mostly off-white to golden, elongate, dense. Trochanters mostly blackish, with some yellow-brown colouring; pile short, very sparse. Femora dark brown to black, with dark marking on dorsal side of the distal end present; pile mostly black, mostly short, dense; ventral pile typically longer, sparse; hind femur with short pile (may have very sparse elongate pile on hind femur) more evenly distributed than on fore and mid femora. Tibiae yellow-brown to dark brown; with mostly short pile, most dense on hind tibia. Tarsi red-brown to dark brown, hind tarsi tend to be darker. ***Wings*** (Fig. 5F). Shape relatively slender; broadest just basal to termination of *CuP* on posterior margin; alula broad; costal margin close to straight, without distinct anteriorly curved flexure; *Sc* termination on *C* aligned with termination of *M_4_* on posterior margin of wing; *R_1_* terminated closer to *R_2+3_* than to *Sc*; termination of *Sc* and *R_1_* well separated; short appendix just beyond fork on *R_4+5_* always absent; cross vein between *M_1_* and *M_2_* absent; cross vein just beyond fork between *R_4_* and *R_2+3_* absent; *R_1_* relatively straight; *R_4_* deep bowing upward; *R_5_* shallow bowing upward; *M_1_* and *M_2_* slightly curved upward; cell *cua* open at margin; *CuA* and *CuP* well separated. Dark marking on *R_1_* positioned just basal to humeral cross vein; membrane without smoky brown infuscation; appearing almost entirely hyaline; isolated darker patches absent. Tuft of pile on base of wing white. Haltere with pale brown to yellow stalk; bulb dark brown.

***Abdomen*** (Fig. 3F). Colour of abdomen generally black; T_2_ with posterior margin stout and relatively broad; abdomen tapering abruptly after T_3_. Tergites with pruinescence largely absent; membrane between T_1_ and T_2_ dark brown to black; medial brown pruinescent vitta indistinct, extending from the posterior margin of T_1_ to terminalia. Pile on tergites mostly black, both long and short, of intermediate density; along anterior margins of T_2_ white to pale yellow, elongate, sparse; posterolateral pile on T_2_–T_4_ black, elongate, dense; T_5_ with pile along lateral margins more evenly distributed than that of T_2_–T_4_. Sternites typically paler than tergites; grey to black; pruinescence silvery, relatively sparse. Sternites with pile mostly black, mostly long, sparse; pile on S_1_ and S_2_ noticeably longer, white; pile on membrane adjacent to lateral margins of S_2_–S_4_ typically with profuse, decumbent, elongate, pale yellow to golden.

***Genitalia*** (Fig. 8E, F). Hypandrium triangular in shape; broad, tapering gradually towards the apex; laterally convex; 2.1× longer than basal width; with apical 1/5 projecting past the top of the gonocoxites. Pile on apical 1/3 of the hypandrium. Gonocoxite apical 1/2 not parallel sided; gonocoxites widest in apical 1/3, narrowing apically; rounded apically. Gonocoxite vestiture on the lateral 1/2, of apical 1/3. Gonostylus narrowed medially; and globular apical section. Phallus near parallel sided; narrowing apically.

**Female**. Same as male, except for genitalia dimorphism and the following characters: ***Head***. Ocellar tubercle width between eyes at the anterior ocellus 3.8–4.5× the length of the anterior ocellus. Frons width anterior to ocellar tubercle 0.8× the width above antennal insertions. ***Wings***. Sharp short appendix just beyond fork on *R_4_* sometimes present.

##### Geographical distribution.

Known from only a single locality on the Hantamsberg near Calvinia (Fig. 1)

##### Biology.

Known to be on the wing in September and has been recorded visiting flowers in the Asteraceae genus *Felicia*. A male and a female were caught mating on a mountain peak, potentially hill-topping (RMCA-ENT 000056705; RMCA-ENT 000056706; RMCA.

##### Etymology.

From the Latin *parva* = little; referring to the small size of this species compared to all other species in this clade. To be treated as a noun in apposition.

## ﻿Discussion

Based on morphological and molecular data, the spring-flying Namaqualand clade of *Prosoeca* (clade D_4_ in Theron et al. 2023) now consists of six species, three of which are newly described here. While a complete revision of *Prosoeca* is desirable, the poor state of the taxonomy has prevented this in the past (Barraclough 2017). Assembling a morphological phylogeny has not been possible, but the recent molecular phylogeny (Theron et al. 2023) has allowed us to revise the genus in a pragmatic, step by step, manner. While the addition of molecular data has resolved some relationships, fresh material is not available for all species (Theron et al. 2023). However, the monophyly of clade D_4_ is supported by molecular data (Theron et al. 2023) and can be identified morphologically by the pale grey ring around the thorax. No species belonging to this clade has been found to be on the wing outside of the spring flowering season and this may represent a unique seasonally restricted radiation of species. While one other undescribed species of *Prosoeca* can be found on the wing in spring in the Greater Cape Floristic Region, it is morphologically distinct (small and grey but without the pale ring on the thorax) from the clade and falls within the sister clade D_3_ in Theron et al. (2023). In addition, there are several undescribed species which can be found in these karoo habitats during other seasons of the year (mainly autumn), but these do not fit the morphological definition of the focal clade provided here. Unfortunately, no fresh material is available for these species to allow confirmation of their placement using molecular data (pers. obs. G.L. Theron).

The low-lying plains of the Knersvlakte region (formerly a palaeochannel of the proto-Orange River) seem to represent an interesting geographical break in species distribution in this group, with no *Prosoeca* species collected from this area during spring. The only species that straddles this geographic break is *P. peringueyi*, which has a strong geographic disjunction across the Knersvlakte (Fig. 1). Interestingly, northern and southern populations of *P. peringueyi* differ morphologically (see wing venation trait discussion in treatment comments) and exhibit a degree of genetic differentiation. As COI differentiation between the northern and southern populations (mean between group distance: 6.27%, range: 5.22%–7.22%; mean within northern group distance: 2.04%, range: 0.15%–5.23%; mean within southern group distance: 2.15%, range: 1.08%–2.76%) was below the 11% threshold generally seen between species in this clade (Table 2), we have chosen not to describe the northern/southern populations as separate species. The fact that both older and more recent divergence events have occurred across the Knersvlakte suggests that this low-lying, arid region may represent a persistent geographic/environmental barrier through the evolutionary history of this *Prosoeca* clade, promoting diversity through repeated allopatric speciation. The concentration of species on the Hantamsberg, and the fact that *P. parva* is only known from this mountain, is another interesting pattern. While this could simply be an artefact of sampling intensity, or a product of concentration of hill-topping individuals of species from the surrounding lower lying areas, it is possible that the cooler higher elevation environment on this mountain have promoted divergence. Interestingly, flowering phenologies of plants on the Hantamsberg are delayed relative to lower lying areas (pers. comm. J. Manning, June 2023) and collections of *Prosoeca* from this area tend to be from later in the season, suggesting the potential role of temporal shifts in emergence times in promoting diversity, although this hypothesis needs more detailed investigation. The greater Succulent Karoo area remains critically under-sampled, particularly outside of the spring flowering season, and possibly harbours more undescribed species.

As with most *Prosoeca* species, the species investigated here have almost all been found foraging on flowers and many specimens had substantial amounts of pollen on their bodies (Fig. 3B). The sparse label data available provides valuable insights to the plant-pollinator interactions these flies may be involved in. For some species however, almost nothing is known about the plants they feed on and more direct field observations would greatly contribute to the knowledge in these systems. While several plant-focused studies have investigated pollination by *Prosoeca* in the region (e.g., Manning and Goldblatt 1996; Goldblatt and Manning 2007; Pauw et al. 2020), these were undertaken with limited knowledge of the fly taxonomy creating uncertainty in inferences about the importance of particular *Prosoeca* species for pollination of the large guild of plants in the region relying on these pollinators. The improved taxonomic resolution of this important group of pollinators we provide here will allow more accurate interpretation of coevolved pollination guilds in Namaqualand, improving conservation potential in the area. Further studies may be needed in cases where voucher material is not available. As is the case for all *Prosoeca* species, the larval hosts are currently unknown, despite attempts to investigate this. Understanding of their life-cycle requirements is essential for ensuring effective conservation of this important group of fly pollinators.

## Supplementary Material

XML Treatment for Prosoeca

XML Treatment for Prosoeca torquata

XML Treatment for Prosoeca marinusi

XML Treatment for Prosoeca peringueyi

XML Treatment for Prosoeca aquilo

XML Treatment for Prosoeca ora

XML Treatment for Prosoeca parva

## ﻿Additional information

### Use of AI

No use of AI was reported.

### Author ORCIDs

Genevieve L. Theron https://orcid.org/0000-0002-1071-9014

Allan G. Ellis https://orcid.org/0000-0001-6310-2870

John M. Midgley https://orcid.org/0000-0003-1203-3750

### Data availability

All of the data that support the findings of this study are available in the main text or Supplementary Information.

## Supplementary materials

Supplementary material 1 A condensed and summarised version of the phylogenetic tree from Theron et al. (2023) Data type eps Explanation note The bar on the right-hand side of the tree indicates the named genera, while the black portion of the bar represents *Prosoeca**sensu lato*. The number of species within a clade is shown in brackets. https://binary.pensoft.net/file/1454277This dataset is made available under the Open Database License (http://opendatacommons.org/licenses/odbl/1.0/). The Open Database License (ODbL) is a license agreement intended to allow users to freely share, modify, and use this Dataset while maintaining this same freedom for others, provided that the original source and author(s) are credited.Genevieve L. Theron, Allan G. Ellis, John M. Midgley
